# Dissecting Acute Drug‐Induced Hepatotoxicity and Therapeutic Responses of Steatotic Liver Disease Using Primary Mouse Liver and Blood Cells in a Liver‐On‐A‐Chip Model

**DOI:** 10.1002/advs.202403516

**Published:** 2024-06-13

**Authors:** Hanyang Liu, Guo Yin, Marlene Sophia Kohlhepp, Fabian Schumacher, Jana Hundertmark, Mohamed I. Abdelwahab Hassan, Felix Heymann, Tobias Puengel, Burkhard Kleuser, Alexander Sandy Mosig, Frank Tacke, Adrien Guillot

**Affiliations:** ^1^ Department of Hepatology & Gastroenterology Campus Virchow‐Klinikum and Campus Charité Mitte Charité – Universitätsmedizin Berlin 13353 Berlin Germany; ^2^ Institute of Pharmacy Freie Universität Berlin Königin‐Luise‐Str. 2+4 14195 Berlin Germany; ^3^ Institute of Biochemistry II Center for Sepsis Control and Care Jena University Hospital 07747 Jena Germany

**Keywords:** fibrosis, liver diseases, macrophages, microfluidic biochip, NAFLD, NASH, steatohepatitis

## Abstract

Metabolic dysfunction‐associated steatotic liver disease (MASLD) is hallmarked by hepatic steatosis, cell injury, inflammation, and fibrosis. This study elaborates on a multicellular biochip‐based liver sinusoid model to mimic MASLD pathomechanisms and investigate the therapeutic effects of drug candidates lanifibranor and resmetirom. Mouse liver primary hepatocytes, hepatic stellate cells, Kupffer cells, and endothelial cells are seeded in a dual‐chamber biocompatible liver‐on‐a‐chip (LoC). The LoC is then perfused with circulating immune cells (CICs). Acetaminophen (APAP) and free fatty acids (FFAs) treatment recapitulate acute drug‐induced liver injury and MASLD, respectively. As a benchmark for the LoC, multiplex immunofluorescence on livers from APAP‐injected and dietary MASLD‐induced mice reveals characteristic changes on parenchymal and immune cell populations. APAP exposure induces cell death in the LoC, and increased inflammatory cytokine levels in the circulating perfusate. Under FFA stimulation, lipid accumulation, cellular damage, inflammatory secretome, and fibrogenesis are increased in the LoC, reflecting MASLD. Both injury conditions potentiate CIC migration from the perfusate to the LoC cellular layers. Lanifibranor prevents the onset of inflammation, while resmetirom decreases lipid accumulation in hepatocytes and increases the generation of FFA metabolites in the LoC. This study demonstrates the LoC potential for functional and molecular evaluation of liver disease drug candidates.

## Graphical abstract

This study demonstrates that a multicellular biochip‐based liver sinusoid model mimics early MASLD pathomechanisms. This liver‐on‐a‐chip may be used to investigate the therapeutic effects of drug candidates, such as lanifibranor and resmetirom. This is shown by direct measurement of blood immune cell recruitment, gene expression analyses, cytokine release, and free fatty acid metabolite measurement.

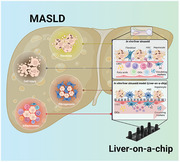

## 1 Introduction

Metabolic dysfunction‐associated steatotic liver disease (MASLD) is a widespread epidemic with a rising incidence and currently affecting ≈30% of the world's population.^[^ ^1^ ^]^ MASLD is an umbrella term that comprises a spectrum of metabolic liver disorders varying in severity of hepatocytic lipid deposition, hepatic injury, inflammation, and fibrosis.^[^ ^2^ ^]^ MASLD may progress from simple steatosis to metabolic dysfunction‐associated steatohepatitis (MASH), cirrhosis, liver failure, and cancer.^[^ ^3^ ^]^ MASLD comorbidities (e.g., arterial hypertension, dyslipidemia, type 2 diabetes) are treated as they occur, and one of the most efficacious approaches remains recommending lifestyle and diet adjustments. This situation reflects a relative lack of knowledge of MASLD pathomechanisms and potent therapeutic targets for intervention.^[^ ^4^ ^]^ Nevertheless, several compounds are undergoing (pre‐)clinical testing and may enlarge the arsenal of therapeutic options offered to MASLD/MASH patients. As such, the pan‐peroxisome proliferator‐activated receptor (PPAR) agonist lanifibranor and the thyroid hormone receptor‐*β* agonist resmetirom are amongst the most promising drug candidates.^[^ ^5^ ^]^ Both molecules have been shown to exert a wide range of therapeutic effects, mostly through the control of metabolic injury in hepatocytes, hepatic stellate cell (HSC) activation, and macrophage polarization.^[^ ^6^, ^7^ ^]^ Despite this medical burden, until very recently no pharmacological agent was approved for the treatment of MASLD. In March 2024, the U.S. Food and Drug Administration (FDA) approved resmetirom (Rezdiffra) for the treatment of adults with MASH with moderate to advanced liver fibrosis. Resmetirom treatment leads to MASH resolution with no worsening of fibrosis, or fibrosis improvement by at least one stage with no worsening of the MASLD activity score in ≈25% patients, in comparison to a placebo response rate of ≈10%.^[^ ^8^ ^]^

Numerous cellular interactions among parenchymal cells and resident or circulating immune cells (CICs) drive the alteration of hepatic homeostasis upon injury.^[^ ^9^ ^]^ Over the course of MASLD progression, which may spread over decades, lipids gradually accumulate in hepatocytes, a phenomenon referred to as steatosis. Metabolic dysfunction, lipotoxicity, cell stress, and inflammation‐mediator secretion occur concomitantly in hepatocytes, which can activate quiescent HSC into fibrogenic myofibroblasts, induce the secretion of numerous chemokines by liver sinusoidal endothelial cells (LSECs), activate liver resident immune cells and recruit CICs. All the above‐mentioned cellular players are encountered at the interface between the blood stream and liver parenchyma and as such, the liver sinusoid microenvironment is regarded as a “hot zone” in the liver, driving MASLD initiation and progressive loss of hepatic functions and increased fibrogenesis and inflammation.^[^ ^10^, ^11^, ^12^, ^13^, ^14^, ^15^ ^]^ The pathophysiological events taking place in the liver sinusoidal microenvironment are considered representative of the primary features associated with MASLD progression and, thus, may hold the key to identifying and testing future therapeutic agents against MASLD/MASH.

Due to these complex multicellular interactions, preclinical drug development in MASLD is oftentimes studied in vivo, in time‐ and resource‐demanding animal models. Hence, multiple in vitro platforms have been developed to recapitulate functionalities and interactions of hepatic cells, including hepatocytes, HSCs, Kupffer cells (KCs), and LSECs. These advances have been achieved with cell‐lines, with primary cells from mice, or using either human primary or induced‐pluripotent stem cells (iPSCs).^[^ ^16^ ^]^ An alternative is precision‐cut liver slices from human (or mouse) liver.^[^ ^17^ ^]^ However, the absence of dynamic perfusion (i.e., shear stress) and CICs represent a major drawback of many current 2D and 3D static culture systems.^[^ ^18^ ^]^ Furthermore, the use of iPSC‐derived technologies remains complex and shows a lack of reproducibility, while the differentiated cells may or may not reflect the varying functional states encountered in an adult, fully mature liver. Yet, for ethical and technical reasons it remains challenging to access primary, healthy, or diseased human tissues.

In this study, we characterized MASLD features and therapeutic responses in a primary mouse cell‐based liver‐on‐a‐chip (LoC) model, which not only supports a co‐culture of the major cell types defining the sinusoidal microenvironment but also allows for the microfluidic perfusion of CICs, free fatty acids (FFAs) and drug candidates. This microfluidic perfusable model can mimic the liver sinusoid by the assembly of distinct compartments: a parenchymal layer, a vascular layer, and a dynamically circulating medium. The LoC model further allowed us to investigate the therapeutic potential of lanifibranor and resmetirom and to highlight previously overlooked direct effects of those compounds on metabolism and inflammation. In addition, to further compare with the in vivo phenotype, mouse liver tissue samples from corresponding acute drug‐induced liver injury and diet‐induced steatosis models were analyzed and confirmed the functional relevance of the LoC.

## 2 Results

### 2.1 Infiltration of Distinct Immune Cell Populations Characterizes Acute and Chronic Liver Injuries In Vivo

**Figure 1:**
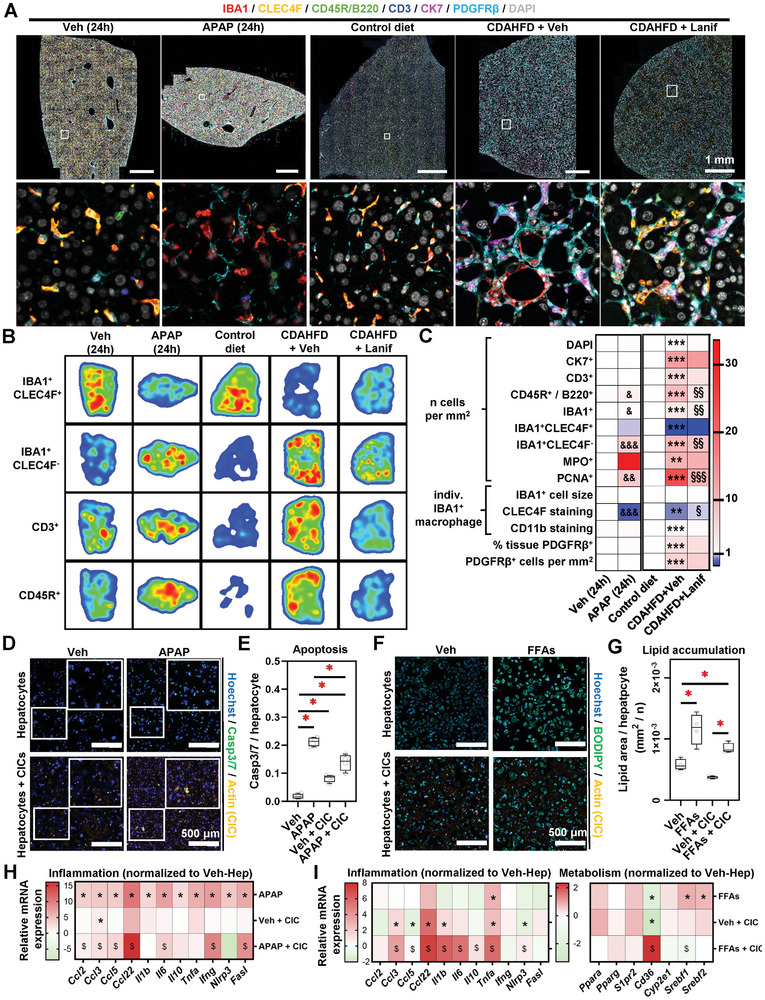
Distinct circulating immune cells are mobilized to the liver and modulate hepatocyte responses to APAP and FFAs. A) Archival formalin‐fixed paraffin‐embedded mouse liver samples were retrieved and subjected to multiplex immunohistochemistry. B) Digital image segmentation and analysis were performed. Heatmaps represent immune cell location in the respective mouse models. C) Imaging cytometry allowed for immune and pathology‐associated cell numbering, and individual cell staining intensity measurements. Individual data points are displayed in Figures S1D,  S2D (Supporting Information. D–I) Conventional 2D cell culture was performed using primary mouse hepatocytes and CICs, exposed to either APAP or FFAs. D) Immunocytochemistry was performed to visualize cell death. Quantification is shown in (E). F) Cells were cultured in the presence of FFAs and BODIPY staining was applied to visualize lipid deposition. G) BODIPY quantitation. H,I) Relative gene expression was analyzed by qRT‐PCR. Sample sizes: A–C) *n*(Veh) = 4, n(APAP) = 4, *n*(control diet) = 4, *n*(CDAHFD + Veh) = 7, *n*(CDAHFD + Lanif) = 7. D–I) *n* = 4 per group. Abbreviations: APAP: acetaminophen; CDAHFD: choline‐deficient, amino acid‐defined high‐fat diet; Lanif: lanifibranor; Veh: vehicle; CK7: cytokeratin 7; CD3: a cluster of differentiation 3; CD45R: cluster of differentiation 45 receptor; IBA1: Ionized calcium‐binding adaptor molecule 1; Ccl: Chemokine (C‐C motif) ligand; CLEC4F: C‐Type Lectin Domain Family 4 Member F; MPO: myeloperoxidase; PCNA: proliferating cell nuclear antigen; CD11b: cluster of differentiation 11b; PDGFRβ: platelet‐derived growth factor receptor beta. C) One‐way ANOVA followed by Tukey's multiple comparison was performed. &*p* < 0.05; &&*p *< 0.01; &&&*p *< 0.005 as compared to vehicle; **p < 0.01; ***p < 0.005 as compared to control diet; §*p *< 0.05; §§*p *< 0.01; §§§*p *< 0.005 as compared to CDAHFD + vehicle. E,G,H,I) One‐way ANOVA followed by Tukey's multiple comparison test was performed. H, I) *p < 0.05 as compared to a vehicle; $*p *< 0.05 as compared to Veh + CIC.

Acetaminophen (APAP) injection in mice causes acute liver failure, while chronic feeding a choline‐deficient high‐fat diet (CDAHFD) causes steatohepatitis in mice.^[^ ^7^ ^]^ Liver samples from APAP‐injected and CDAHFD‐fed mice were analyzed by multiplex immunostaining to assess the spatial distribution of multiple cell populations within the liver (**Figure** **1**A; Figures S1,  S2, Supporting Information). APAP‐ and CDAHFD‐exposed mice displayed a profound infiltration of monocyte‐derived macrophages (IBA1^+^CLEC4F^−^) associated with a reduction in the resident liver macrophage pool (i.e., Kupffer cells, IBA1^+^CLEC4F^+^). Furthermore, B lymphocytes accumulated in mouse livers in both models and T cells in the CDAHFD model. Indeed, a quantitative analysis revealed a statistically significant increase in B cells (CD45R/B220^+^), total macrophages (IBA1^+^), monocyte‐derived macrophages (IBA1^+^CLEC4F^−^), and cycling hepatocytes (PCNA^+^) in APAP‐injected mouse livers (Figure S1D–F, Supporting Information). In addition to this, biliary cells (CK7^+^), T cells (CD3^+^), myeloid cells (CD11b^+^), neutrophils (MPO^+^), and HSC (PDGFRβ^+^) numbers increased in CDAHFD‐fed mice (Figure S2D–F, Supporting Information). This data confirmed the broader elevation of immune infiltration and cellular proliferation associated with a reduction of liver resident macrophages marked in mouse fatty livers. Importantly, treatment with the pan‐peroxisome proliferator‐activated receptor (PPAR) agonist lanifibranor in the MASLD model significantly lowered the accumulation of CD45R/B220^+^, IBA1^+^, IBA1^+^CLEC4F^−^ and PCNA^+^ cells, while neither affecting PDGF‐Rβ^+^ cell accumulation nor IBA1^+^ cell staining intensities for CCR2 and CD11b (Figure 1B,C; Figure S2D–F, Supporting Information).^[^ ^19^ ^]^

As these data emphasized a potent infiltration of leukocytes in the liver upon those injury models in vivo,^[^ ^20^ ^]^ we assessed the potential implications of CICs on APAP‐ and FFA‐exposed primary mouse hepatocytes in vitro, focusing on hepatocyte cell death (APAP) or steatosis (FFA). We first aimed at characterizing primary hepatocyte survival and metabolic functions after isolation and over an extended culture of up to 120 h. First, we seeded the hepatocytes on a conventional 2D culture dish and measured mitochondrial activity using an MTS assay. Figure S3A (Supporting Information) shows that mitochondrial activity gradually decreases after 72 h, either reflecting a decreasing number of cells per well (i.e., cell death) or a loss of metabolic functions. We next performed a 7‐AAD staining, alongside counting total hepatocyte numbers, and could evidence that there is an increasing number of cells engaging into cell death, although the overall number of cells is increasing over time (Figure S3B,C, Supporting Information). Next, we sought to assess hepatocyte metabolic functions. We could evidence that most enzyme gene expressions were altered from 72 h in culture, while APAP‐induced hepatocyte toxicity, which relies on hepatocyte metabolic functions to generate the toxic metabolites, is maintained over 96 h of culture (Figure S3C,D, Supporting Information).^[^ ^21^ ^]^

We next sought to investigate the influence of CICs on hepatocyte response to APAP and FFAs. The co‐culture of primary hepatocytes with CIC induced hepatocyte apoptosis at a moderate level (Figure 1D,E). APAP treatment led to a significantly stronger increase in hepatocyte cell death. Intriguingly, hepatocyte apoptosis was reduced when cells were exposed to APAP in the presence of CICs, as compared to APAP treatment alone (Figure 1D,E). This could be attributed to a reduced APAP clearance in the culture medium from hepatocytes cultured with CICs as compared to hepatocytes alone, associated with an apparent reduced in CYP2E1 immunostaining in culture (Figure S3E,F, Supporting Information). FFA treatment increased hepatocyte lipid accumulation at a similar level in the presence or absence of CICs (Figure 1F,G). Next, we evaluated inflammation‐related gene expressions (*Ccl2*, *Ccl3*, *Ccl5*, *Ccl22*, *Il1b*, *Il6*, *Il10*, *Tnfa*, *Ifng*, *Nlrp3* and *Fasl*) in this set‐up. In comparison to vehicle groups, all these factors were upregulated by APAP, while CIC addition alone to the culture increased the expression levels of *Ccl3*. The combination of CICs and APAP further increased the expression of *Ccl2*, *Ccl3*, *Ccl5*, *Ccl22*, *Ifng*, and *Fasl* as compared to CIC addition alone (Figure 1H; Figure S3G, Supporting Information). Furthermore, inflammation‐ and metabolism‐related (*Ppara*, *Pparg*, *S1pr2*, *Cyp2e1*, *Srebf1*, and *Srebf2*) gene expression was assessed in FFA‐treated hepatocytes. In comparison to the vehicle (Veh) group, increased expression levels of *Tnfa, Srebf1*, and *Srebf2* were found for inflammatory genes under FFA treatment. In the presence of CICs, the expression of *Ccl22* and *Tnfa* were upregulated. In addition, CIC + FFA treatment enhanced the expression of *Ccl3*, *Ccl22*, *Il1b*, *Il6*, *Tnfa*, and *Nlrp3* on hepatocytes, as compared to CIC + Veh. Amongst the metabolism‐related genes, *Cd36* showed a marked increase in the FFA+CICs condition as compared to Veh + CICs, as opposed to a decrease in FFA or CIC addition alone, as compared to control hepatocytes (Figure 1I; Figure S3H, Supporting Information). Taken together, both in vivo and in vitro data demonstrate that not only liver residents but also CICs play potent roles in modulating the liver response to acute drug‐induced and chronic steatotic liver injuries.

### 2.2 Setting‐Up the LoC Model to Recapitulate Acute Liver Injury

**Figure 2:**
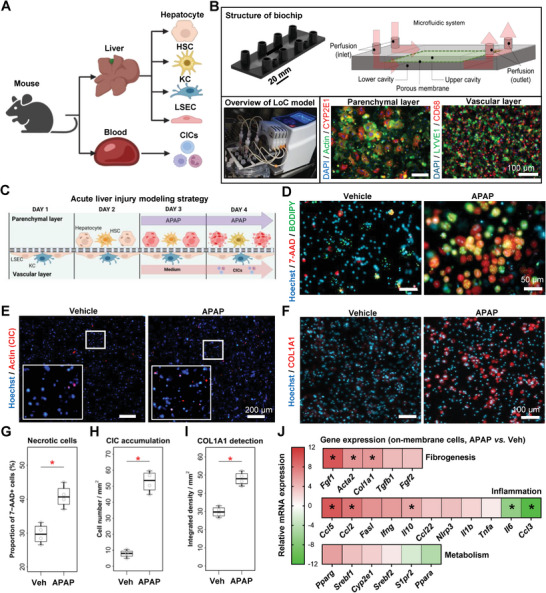
Designing and validating a microfluidically perfused LoC for acute liver injury modeling. A) Schematic view of the liver and circulating cells isolated prior to LoC seeding. B) LoC set‐up depicting liver cell organization into the LoC during CIC perfusion. The upper image depicts the biochip used in this model. The lower left photograph shows the experimental set‐up within the cell culture incubator, including a peristaltic pump. The lower right microscopy images show the two layers of cells, staining by immunocytochemistry for the indicated markers. C) Experimental outline of the acute APAP injury model in the LoC. Fluorescence microscopy was used to visualize D) cell death (parenchymal layer), E) labeled CIC attachment to the membrane (vascular layer), and F) collagen 1A1 expression (parenchymal layer); quantitation is shown in (G–I), respectively. J) Total mRNA was extracted from the membrane at the end of the experiments, and gene expression was analyzed. Data shows the relative gene expression in APAP‐treated LoC as compared to vehicle. Individual points are depicted in Figure 5A–C (Supporting Information). Sample sizes: B–J) *n* = 4 per group. Abbreviations: APAP: acetaminophen; CICs: circulating immune cells; Ccl: Chemokine (C‐C motif) ligand; COL1A1: collagen, type I, alpha 1; GM‐CSF: granulocyte‐macrophage colony‐stimulating factor; HSC: hepatic stellate cell; IL: interleukin; IFN: interferon; KC: Kupffer cell; LSEC: liver sinusoidal endothelial cell; Tnfa: tumor necrosis factor‐alpha; Veh: vehicle. Paired student's *t*‐tests were performed. *p < 0.05 as compared to vehicle.

To decipher cell type‐specific pathogenic contributions including the roles of CICs in vitro, we aimed at designing a multicellular and dynamic cell culture model of the hepatic sinusoid that is entirely based on primary cells (from mouse liver and blood). First, we optimized a protocol for multiple primary mouse liver cell isolation (hepatocytes, HSCs, KCs, and LSECs) and mouse blood CICs. The purity of each cell population was verified by morphological characters or/and immunofluorescence staining in singular cell cultures (**Figure** **2**A; Figure S4, Supporting Information). Primary cells were then seeded onto the biochip to generate the LoC, in which a “parenchymal layer” of hepatocytes and HSCs was separated from the “vascular layer” that consists of LSECs and KCs by a porous membrane (Figure 2B). Based on the findings from above, hepatocytes were reliably maintained in culture for a total period of 72 h after isolation. To verify the relevance of the LoC to study acute injuries, APAP was added for 24 h to the perfused culture medium after cell seeding and actin‐labelled CICs were perfused for the last 30 min of the experiments (Figure 2C). Cells either from the biochip membrane or from the perfusate medium were harvested for further analyses. We then measured in the LoC hepatocyte cell death, CIC recruitment, and collagen immunostaining (Figure 2D–F). In the LoC, APAP exposure promoted cell necrosis (7‐AAD^+^) (Figure 2D,G), CIC migration from the perfusate to the chip (Figure 2E,H), and increased COL1A1 expression (COL1A1^+^) despite the short treatment time (Figure 2F,I). Inflammation‐ (*Ccl5*, *Ccl2* and *Il10*) and fibrogenesis‐related (*Fgf1*, *Acta2*, and *Col1a1*) gene expressions were significantly upregulated, while expressions of *Il6* and *Ccl3* were downregulated (Figure 2J; Figure S5A–C, Supporting Information). These data show that the LoC can be used to model APAP‐induced cellular damage, immune cell recruitment, and initiation of fibrogenesis.

To further explore the added value of the LoC as compared to conventional culture models, APAP treatment was applied on mono‐ or bi‐cellular 2D cultures of liver primary cells (hepatocytes, HSCs, and LSECs + KCs). This experiment showed that hepatocytes were the only cells directly responding to APAP by showing an increased Apopxin signal (Figure S6A,B, Supporting Information). Similarly, hepatocytes were the only cells to accumulate significant levels of lipids when exposed to FFAs (Figure S6C, Supporting Information). Gene expression analysis was performed on HSCs and LSECs to respectively measure the expression of fibrogenesis‐related genes and adhesion molecules. Results indicated that APAP significantly upregulated expression of *Fgf1*, and *Tgfb1* in HSCs (Figure S6D, Supporting Information). FFA treatment alone also induced the expressions of *Acta2*, *Fgf1*, and *Tgfb1* in primary HSCs. APAP did not cause any significant changes in adhesion molecule gene expressions compared to vehicles, whereas lanifibranor seemed to reduce the expression of *Icam1* and increase that of *Vcam1* in LSECs (Figure S6E, Supporting Information). In summary, the results from 2D cell culture confirmed that APAP mostly led to hepatocyte toxicity, while the contribution of multiple liver cell types seems necessary to drive the complex inflammatory and fibrogenic responses observed in the LoC and in vivo.

### 2.3 Recapitulation of MASLD and Therapeutic Effects of Lanifibranor in the FFA‐Challenged LoC Model

We next aimed at investigating the potential use of an FFA‐based MASLD model to evaluate cellular pathogenic interactions and the therapeutic potential of drug candidates. Liver response to lipid accumulation is the result of signal integration by multiple cell types.^[^ ^22^ ^]^ As an initial step, mono‐ or bi‐culture models showed that hepatocytes were the only cells directly responding to FFA treatment by exhibiting increased apoptosis and intracellular lipid droplet accumulation (Figure S6A–C, Supporting Information). Hepatocellular apoptosis and lipid accumulation were not significantly changed by lanifibranor in primary hepatocytes. FFA‐exposed HSCs on the other hand, were marked by an increased gene expression of *Acta2*, *Fgf1*, and *Tgfb1* compared to vehicles, which was not significantly changed in the presence of lanifibranor (Figure S6D, Supporting Information). Additionally, and when applied to LSECs, FFAs significantly reduced the expression of *Icam1* compared to vehicle, while the presence of lanifibranor significantly upregulated the expression of *Vcam1* compared to FFAs (Figure S6E, Supporting Information). Thus, the results from static cell culture confirmed that hepatocytes accumulate intracellular lipid vesicles when exposed to FFAs in our conditions. Moreover, the presence of other cell types is required to mimic the overall tissue response to specific stimuli through additional, hepatocyte‐independent mechanisms.

**Figure 3:**
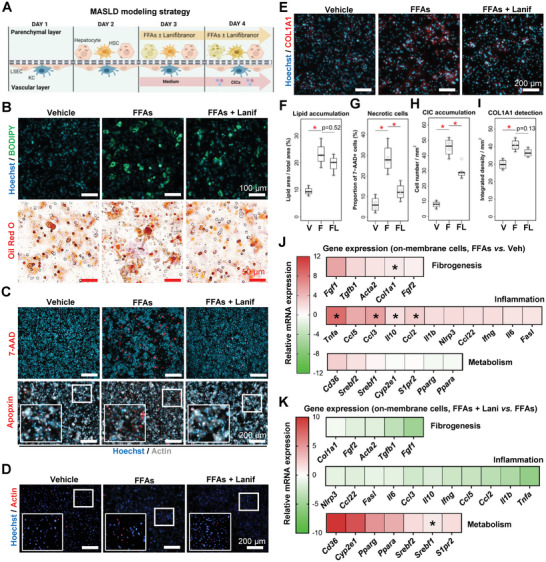
The LoC for modeling key features of MASLD and therapeutic effects of lanifibranor. A) Experimental outline of the FFA‐induced MASLD model in the LoC. B) Lipid vesicle accumulation in LoC hepatocytes was verified by BODIPY and Oil Red O staining. C) Cell death was visualized by 7‐AAD and Apopxin staining, D) immune cell recruitment by using reporter CICs, and E) collagen accumulation by immunostaining. B–E) Parenchymal layer. F–I) Staining quantitation from the staining displayed in panels (B–E). Relative gene expression was assessed from the LoC membrane, and is displayed either as J) FFA‐treated relative to vehicle, or K) FFA plus lanifibranor relative to FFA plus vehicle. Individual points are depicted in Figure S7A–C (Supporting Information). Sample sizes: B–K) *n* = 4 per group. Abbreviations: COL1A1: collagen, type I, alpha 1; CICs: circulating immune cells; F or FFA: free‐fatty acid treated; FL or FFAs + Lanif: free fatty acid plus lanifibranor; V or Veh: vehicle. Paired student's *t*‐tests were performed. *p < 0.05 as indicated or as compared to vehicle.

We next adapted the LoC to investigate the involvement of multiple cell types during MASLD. Following cell seeding, FFAs were introduced to the LoC to induce steatosis. Simultaneously, lanifibranor was applied to evaluate its therapeutic potential to modulate the degree of MASLD hallmarks (**Figure** **3**A). Intracellular lipid deposition, cell death, CIC recruitment, and collagen deposition were visualized by immunostaining (Figure 3B–E). The lipid vesicle staining (Oil Red O and BODIPY) showed that lipid accumulation was effectively increased by FFAs (Figure 3B). The quantification only showed a trend to a reduced lipid accumulation, when lanifibranor was added to the culture (Figure 3F). Hepatocytic necrosis (7‐AAD^+^), apoptosis (Apopxin^+^) (Figure 3C), and the proportion of necrotic cells (Figure 3G) were shown to be fuelled by FFAs, and cell death was significantly prevented by lanifibranor. CICs (Actin^+^) recruitment to the LoC was promoted by FFAs and ameliorated by the lanifibranor treatment (Figure 3D,H). Collagen production (COL1A1^+^) was similarly increased by FFAs and tended to be reduced by lanifibranor, although not significantly (Figure 3E,I). The quantitative gene expression analysis on membrane‐adhered cells of the LoC showed that the expression of inflammatory (*Tnfa*, *Ccl3*, *Il10*, and *Ccl2*) and fibrogenic (*Col1a1*) factors were significantly upregulated by FFAs (Figure 3J; Figure S7A–C, Supporting Information). In contrast, the expression levels of metabolic factors (*Cyp2e1*, *Pparg*, *Ppara*, *Srebf2*, *Srebf1*, and *Cd36*) were upregulated by the PPAR agonist lanifibranor, while the expression of inflammatory factors (*Il10*, *Ccl2*, *Il1b*, and *Tnfa*) and of the fibrogenic factor *Fgf1* was repressed by lanifibranor as compared to FFA + vehicle (Figure 3K; Figure S7A–C, Supporting Information). Taken together, FFAs induce steatosis, inflammation, and fibrogenesis to recapitulate key features of MASLD in the LoC. Furthermore, the LoC allowed us to dissect the effects of lanifibranor in augmenting protective metabolic pathways and reducing lipotoxicity, as well as the subsequent inflammation and fibrogenesis.

### 2.4 Lanifibranor Prevents Circulating Inflammatory Cytokine Secretion in the LoC Injury Models

**Figure 4:**
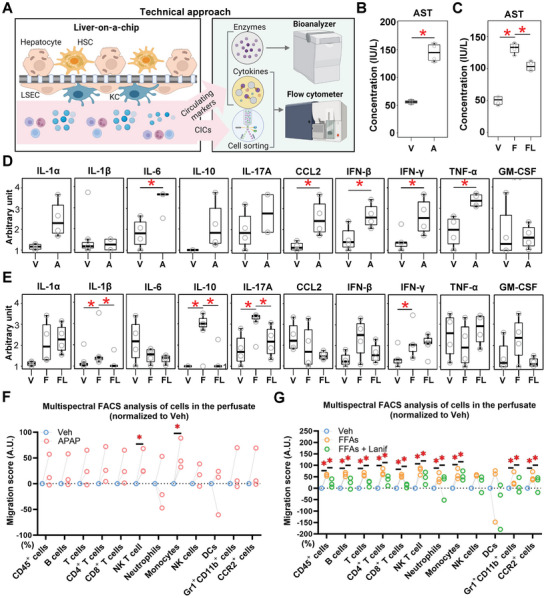
Lanifibranor treatment reduces hepatocyte injury, inflammatory cytokine, and circulating immune cell recruitment in the LoC. A) Wild‐type CICs were perfused for 30 min into the LoC after APAP‐, FFA‐, or FFA and lanifibranor treatment, and the remaining cells from the perfusate were analyzed by multispectral flow cytometry. The dynamically perfused culture medium may be collected at the end of the experiment and used for further measurements including cytokine and transaminase measurement. AST activity was measured in the LoC exposed to B) acetaminophen, or C) FFAs and lanifibranor. Circulating cytokine levels measured in the perfusate from the LoC exposed to D) acetaminophen, or E) FFAs and lanifibranor. CIC depletion (migration score) from the LoC perfusate after F) APAP or G) FFA with or without lanifibranor treatment is shown for the respective immune cell types. Relative changes in absolute cell numbers as compared to the appropriate vehicle conditions. Sample sizes: B–E) *n* = 4 per group, F,G) *n* = 3 per group. Abbreviations: A or APAP: acetaminophen; AST: aspartate aminotransferase; CCL2: Chemokine (C‐C motif) ligand 2; CICs: circulating immune cells; DCs: dendritic cells; F or FFAs: free fatty acids; FL or FFAs + Lanif: free fatty acids plus lanifibranor; FACS: fluorescence‐activated cell sorting; GM‐CSF: granulocyte‐macrophage colony‐stimulating factor; HSCs: hepatic stellate cells; IFN: interferon; IL: interleukin; IU: international units; KCs: Kupffer cells; LSECs: liver sinusoidal endothelial cells; NK: natural killer; TNF‐*α*: tumor necrosis factor‐alpha; V or Veh: vehicle. One‐way ANOVA followed by Tukey's multiple comparison test and paired student's *t*‐tests were performed. *p < 0.05 as indicated or as compared to vehicle.

MASLD as well as other types of liver injuries are conditions affecting multiple organs, marked by potent elevation in circulating inflammatory biomarkers.^[^ ^23^ ^]^ The experimental set‐up of the LoC allows for the collection of the culture medium circulating through the perfusion system, herein referred to as the perfusate, for further analyses (**Figure** **4**A). APAP treatment resulted in a robust elevation of AST activity in the perfusate (Figure 4B). Similarly, FFAs elevated AST levels compared to the vehicle condition in the LoC perfusate, which was significantly reduced in the presence of lanifibranor (Figure 4C). ALT levels from all groups were below the threshold of detection (data not shown). Furthermore, cytokine levels were measured in the LoC and on conventional co‐culture (hepatocytes +/‐ CICs) exposed to APAP (Figure 4D; Figure S8A, Supporting Information). CCL2, IFN‐*β*, IFN‐*γ*, and TNF‐*α* concentrations were significantly increased by APAP in the LoC (Figure 4D). Noteworthy, IL6 protein levels in the cell culture medium were increased, although gene expression was reduced (Figure 2J). Some discrepancies were observed between conventional and LoC systems; in particular, CCL2 levels seemed to be decreased after APAP exposure in conventional culture, whereas it was increased in the multicellular LoC (Figure 4D; Figure S8A, Supporting Information). The addition of CICs to conventional hepatocyte culture did not affect the cytokine levels, except for IL‐1A levels that were significantly increased, and IL‐6, IL17A, and IL‐12p70 were decreased. Similar to the APAP experiments performed on conventional culture, the FFA and lanifibranor testing did not suffice to generate relevant cytokine level modulation in hepatocytes, while the levels of IL‐1A were increased in all conditions (Figure S8B, Supporting Information). In contrast, cytokine measurement in the LoC indicated that the secretion of IL‐1*β*, IL‐10, IL‐17A, and IFN‐*γ* was significantly enhanced by FFAs, while the secretion of IL‐1*β*, IL‐10, and IL‐17A was significantly lowered by lanifibranor (Figure 4E). These results indicate potent changes in experimental read‐outs allowed by the nature of the LoC and highlight potent inflammatory processes that require dynamic perfusion of CICs in the presence of multiple liver cell types.

### 2.5 Lanifibranor Effectively Reduces CIC Recruitment to the Injured Hepatocytes in the LoC‐MASLD Model

The microfluidic perfusion system in the LoC allows for a direct assessment of immune cell mobilization to the injured sinusoid model. Noteworthy, we observed that the passive diffusion of CICs to the LoC was gradually increasing over time and reached significant values after a 30‐minute perfusion (Figure S9A,B, Supporting Information). We also demonstrate that in our culture conditions, CIC survival after 24 h in conventional culture dishes is ≈90%, while CIC survival in low‐adherence culture flasks is improved (Figure S9C,D, Supporting Information). Additionally, the CCK8 assay performed on CICs perfused in an empty LoC shows a 10% and a 40% drop of absorbance after a 30‐minute or 24‐hour perfusion, respectively (Figure S10E, Supporting Information). This observation ensured that a 30‐minute perfusion was the most appropriate time for characterizing CIC migration to the control and injured LoC.

After a 30 minute‐perfusion, CICs remaining in the circulation were harvested and analyzed by multispectral flow cytometry (Figure 4A). Flow cytometry analysis was based on a 13‐antibody panel (plus live/dead staining), which enables a distinct classification of 12 viable immune cell populations (Figure S10A, Supporting Information). The proportion of each cell population from all experimental groups is displayed to summarize the CIC content in the fresh blood cell preparation and in the post‐perfusion circulation (Figure S10B, Supporting Information). Furthermore, cell numbers were compared among different groups to determine cell migration from the perfusate to the chip membrane. In principle, all immune cell types were significantly migrating out of the perfused medium toward the LoC, yet at different rates and with an apparent faster accumulation of myeloid cells into the LoC without any injury inducers (Figure S10C, Supporting Information). Only the migration of NKT cells and monocytes showed a significant increase after APAP treatment in the LoC (Figure 4F). Comparably, in the MASLD‐like setting, the majority of immune cell populations (CD45^+^, B, T, CD4^+^ T, CD8^+^ T, NKT, NK cells, monocytes, and neutrophils) showed increased migration to the LoC when FFA was applied. Notably, the migration of CICs was reduced upon lanifibranor treatment in comparison to FFA alone (Figure 4G). Importantly, no increased CIC death was detected in the perfusate, and CICs perfused in a LoC containing hepatic cells had a lower cell death rate than cells perfused in an empty chip (Figure S10D, Supporting Information).

We further established a human cell line‐ and primary blood human cell‐based system recapitulating the main features of the mouse LoC herein described (Figure S11, Supporting Information). In this system, we demonstrated the potential of the human LoC (hLoC) to mimic steatosis and inflammation, as well as the therapeutic potentials of lanifibranor, similarly to the mouse cell‐based approach. In order to further characterize CIC accumulation‐related processes from the membrane‐adhering cells in the mouse LoC, gene expression analysis was performed on several adhesion molecules (*Icam1*, *Vcam1*, and *Pecam1*) and immune cell markers (*Cd4*, *Cd8*, *Ccr2*, *Cd68*, and *Clec4f*). Results illustrated that the expression of adhesion molecules was downregulated by APAP treatment (significantly for *Icam1* and *Pecam1*), while the expression of immune cell markers tended to be upregulated although not significantly (Figure S12A,B, Supporting Information). Contrastingly, the expression of adhesion molecules and immune cell markers tended to be upregulated by FFA treatment (significantly for *Icam1*, *Cd4*, and *Ccr2*, while the expression of *Vcam1* was prevented by lanifibranor (Figure S12C,D, Supporting Information), and other immune cell‐related markers showed trends of reduction. In summary, acute APAP‐induced cell toxicity and FFA‐induced steatosis can directly influence CIC mobilization and provoke robust migration from the perfusate to the LoC, whereas lanifibranor can prevent FFA‐induced CIC migration from the circulation to the parenchymal hepatic compartment.

### 2.6 The LoC Maintains Hepatocyte Metabolic Activity and Lanifibranor Moderately Affects Circulating FFA Metabolite Levels in the LoC‐MASLD Model

**Figure 5:**
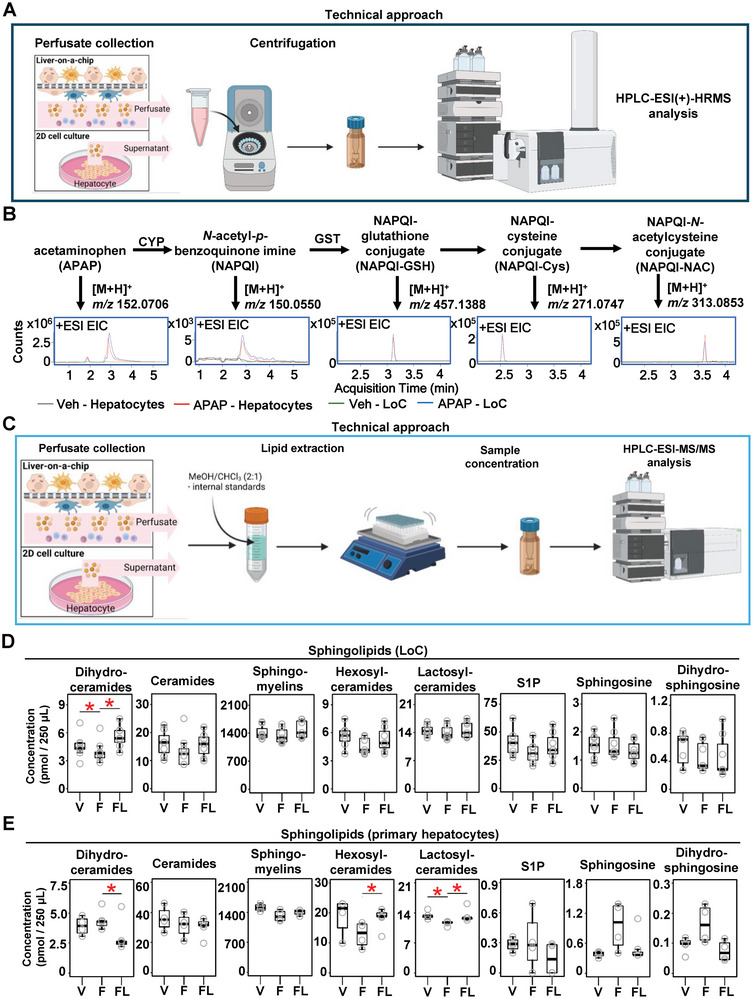
Lanifibranor moderately affects sphingolipid metabolism and release in vitro. A) HPLC‐ESI(+)‐HRMS was used to measure APAP metabolites on B) 2D hepatocyte culture and LoC. C) HPLC‐ESI‐MS/MS was used to measure FFA metabolites. Sphingolipids were measured on D) LoC perfusate, and E) conventional primary hepatocyte culture supernatant. Sample sizes: B,D,E) *n* = 4 per group. Abbreviations: APAP: acetaminophen; CYP: cytochrome P450; EIC: extracted ion chromatogram; F: free‐fatty acid treated; FL: free fatty acids plus lanifibranor treated; GST: glutathione *S*‐transferase; HPLC‐ESI‐HRMS: high‐performance liquid chromatography‐electrospray ionization high‐resolution mass spectrometry; HPLC‐ESI‐MS/MS: high‐performance liquid chromatography‐electrospray ionization tandem‐mass spectrometry; S1P: sphingosine‐1‐phosphate; V: vehicle. Paired student's *t*‐tests were performed. *p < 0.05 as indicated or as compared to vehicle.

We next sought to validate that hepatocytes maintained their metabolic activity when seeded into the multicellular LoC. For this, we collected the perfused medium from the LoC and measured APAP and APAP metabolites by semi‐quantitative mass spectrometry (**Figure** **5**A). We demonstrated that the LoC exposure to APAP led to the presence of APAP metabolites, similarly to primary, conventional hepatocyte cultures (Figure 5B). In order to assess the functional relevance of the LoC‐MASLD model on metabolic features, we measured cholesterol and triglyceride levels in the perfusate, and our data showed no changes whether FFAs or lanifibranor were added to the LoC (Figure S13A, Supporting Information).

We next aimed at dissecting the effects of lanifibranor on FFA metabolite levels in the LoC. For this, we used mass spectrometry to measure sphingolipids, known metabolites with potent effects on the MASLD course (Figure 5C; Figure S13B, Supporting Information).^[^ ^24^ ^]^ Our data showed that only dihydroceramide levels were increased in the FFA + lanifibranor group, whereas no changes were observed in the other groups in the LoC (Figure 5D). Contrastingly, lanifibranor decreased dihydroceramide and restored hexosylceramide and lactosylceramide levels in conventional hepatocyte culture (Figure 5E). Overall, this data indicates an impact of immune and other non‐parenchymal cells not only on inflammatory and fibrogenic but also on metabolic responses during lipotoxicity.

### 2.7 Validation of Therapeutic Responses to Resmetirom in the LoC‐MASLD Model

**Figure 6:**
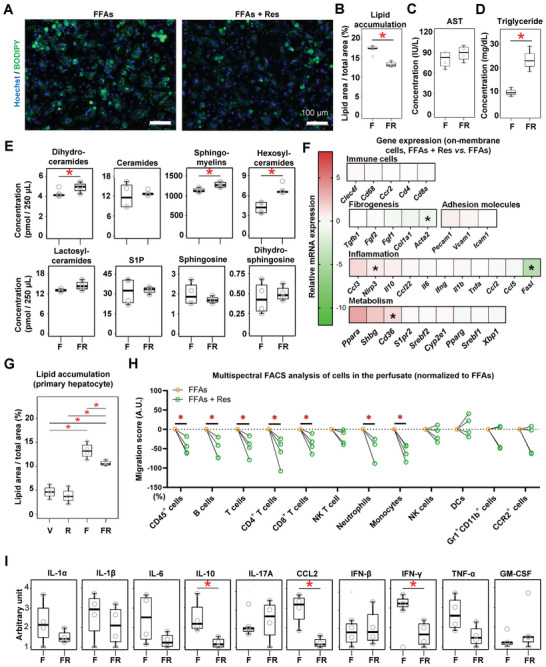
Therapeutic effects of resmetirom on metabolism, inflammation and fibrogenesis in the MASLD‐LoC model. Liver cells were seeded in the LoC as shown in Figure 3A, but using resmetirom instead of lanifibranor. CICs were only perfused for the last 30 min of the experiment. A) BODIPY was used to measure lipid vesicle accumulation in the parenchymal layer, and quantitation is displayed in (B). C) AST activity and D) triglyceride levels were measured in the LoC perfusate with a Bioanalyzer. E) FFA metabolites from the LoC perfusate were analyzed by HPLC‐ESI‐MS/MS. F) Gene expression analysis of the LoC treated with resmetirom or vehicle, shown as relative expression to FFA‐treated LoC. Individual points are depicted in Figure 12D,F,G,I,J (Supporting Information). G) Lipid accumulation was measured in conventional cell culture supernatant of primary hepatocytes exposed to resmetirom, by using BODIY. H) Multispectral flow cytometry was performed to estimate CIC migration to the LoC. I) Circulating cytokine levels in the LoC perfusate were assessed. Sample sizes: *n* = 4 per group. Abbreviations: AST: aspartate aminotransferase; CCL2: Chemokine (C‐C motif) ligand 2; CICs: circulating immune cells; DCs: dendritic cells; F or FFAs: free fatty acids; FR or FFAs + Res: free fatty acids plus resmetirom; NK: natural killer; IL: interleukin; IFN: interferon; S1P: sphingosine‐1‐phosphate; TNF‐*α*: tumor necrosis factor‐alpha; GM‐CSF: granulocyte‐macrophage colony‐stimulating factor. One‐way ANOVA followed by Tukey's multiple comparison test and un‐/paired student's *t*‐tests were performed. *p < 0.05 as indicated.

Building on our methodological and conceptual findings using lanifibranor, we aimed to evaluate the mechanisms of action of the currently only FDA‐approved treatment for MASLD, the thyroid‐hormone receptor‐*β* agonist resmetirom.^[^ ^8^, ^25^ ^]^ Resmetirom significantly reduced lipid accumulation in the FFA‐challenged LoC, as shown by BODIPY staining (**Figure** **6**A,B). Similarly, resmetirom significantly reduced hepatocellular lipid accumulation in conventional 2D hepatocyte culture (Figure S14A, Supporting Information). No changes were observed in AST activity in the LoC perfusate (Figure 6C), whereas circulating triglyceride levels increased in the presence of resmetirom (Figure 6D). Cholesterol levels in the perfusate showed no changes with the administration of FFAs and resmetirom on the LoC (Figure S14B, Supporting Information) Importantly, we demonstrate that resmetirom increased the levels of dihydroceramides, sphingomyelins, and hexosylceramides in the LoC (Figure 6E), whereas singular hepatocyte culture displayed increased sphingolipid salvage pathway as shown by increased S1P and sphingosine, and decreased sphingomyelin, hexosylceramide, and ceramides (Figure S14C, Supporting Information). Comparably, metabolism‐related gene expression changes did not reach statistical significance in the LoC except for an increased *Cd36* expression, although *Pparg*, *Shbg*, and *Xbp1* levels were increased in singular hepatocyte culture (Figure 6F; Figure S14D,E, Supporting Information). Nonetheless, primary hepatocyte culture showed increased intracellular lipid accumulation that was prevented by resmetirom similar to the LoC (Figure 6G), highlighting that despite showing comparable endpoints, distinct mechanisms may be taking place in single versus multiple cell culture systems. Furthermore, inflammation and immune cell‐related gene expression analysis showed increased *Nlrp3* and decreased *Fasl* expression in the LoC whereas hepatocyte culture showed increased *Ccl3*, *Ccl5*, *Ccl22*, and *Ifng* levels (Figure 6F; Figure S14F–H, Supporting Information). There was no change in adhesion molecule and fibrogenesis‐related gene expressions in the LoC (Figure S14I,J, Supporting Information).

Furthermore, immune cell mobilization was assessed by flow cytometry performed on the cells circulating in the LoC perfusate. The proportion of each cell population from all experimental groups is displayed to summarize the CIC content in the post‐perfusion circulation (Figure S14K, Supporting Information). In comparison to FFAs alone, the addition of resmetirom tended to attenuate immune cell depletion/migration (significantly for CD45^+^, B, T, CD4^+^ T, CD8^+^ T cells, neutrophils, and monocytes) (Figure 6H). In addition, resmetirom significantly ameliorated the increased levels of IL‐10, CCL2, and IFN‐*γ* in the LoC perfusate (Figure 6I). Taken together, our data highlights that resmetirom, as a selective thyroid hormone receptor‐*β* agonist, enhanced lipid metabolism in the LoC model. Particularly, resmetirom can attenuate steatosis‐induced inflammation by reducing CIC migration and inflammatory cytokine secretion and exerts potent effects on FFA metabolism that rely on the implication of multiple cell populations beyond hepatocytes.

## 3 Discussion

In this study, we first evidenced the profound changes in the immunological milieu occurring in mouse models of acute drug‐induced and MASLD liver injury models. Then, we describe the relevance and potential applications of a biochip‐based multicellular perfusable in vitro model of acute APAP‐induced liver injury and MASLD, using primary mouse liver and blood cells. Following the optimized isolation of viable primary mouse hepatocytes, HSCs, KCs, LSECs, and CICs, we demonstrated that those cells may be seeded in a two‐layer set‐up, with CIC perfusion on the sinusoidal‐like side of the chip (containing KCs and LSECs). Drug‐induced liver injury‐ and MASLD‐associated characteristics such as cell death, lipid vesicle accumulation, fibrogenesis‐ and inflammation‐related gene expression increase as well as an elevation of circulating inflammatory cytokine levels were recapitulated in this model. Furthermore, we provide results on the use of the LoC for therapeutic candidate testing, here lanifibranor and resmetirom, showing those compounds effectively prevent the onset of inflammation and the accumulation of lipid vesicles in primary hepatocytes. Finally, we elaborate on the consequences of lanifibranor and resmetirom in the regulation of sphingolipid metabolism.

In vitro, methods that can be applied for the study of liver diseases, and particularly for MASLD, are being actively developed.^[^ ^16^ ^]^ A growing number of LoC systems have been developed and some show great promises for large‐scale toxicity assays.^[^ ^26^ ^]^ A crucial advantage of the LoC we herein report is the possibility to dynamically perfuse CICs. Indeed, ongoing tissue injury generally affects a plethora of CICs in vivo, which then drastically affects the disease course. Accordingly, LoC‐derived findings were compared to the respective in vivo models of liver injury and conventional cell culture systems, an approach that allowed us to highlight the advantages and limitations of the LoC. We successfully induced acute APAP‐mediated hepatocyte injury and FFA‐mediated early MASLD‐like features, as described above. By flow cytometry, we could further elaborate on the ability of the LoC to retain specific CIC populations. Interestingly, those data showed some discrepancies with in vivo findings. Those differences may be explained by the inherent properties of the LoC and highlight the limitations of both in vitro and in vivo models of human liver diseases.^[^ ^27^, ^28^ ^]^ Indeed, in our set‐up the LoC may only be used to study acute phenomena, meaning occurring within a 3‐day window, due to the versatile nature of primary cells. Furthermore, the LoC is here devoid of adipose tissue‐derived signals, which impact circulating lipid levels and drive MASLD.^[^ ^29^ ^]^ Importantly, the LoC is devoid of CIC mobilization or renewal from the bone marrow, which should be considered carefully when comparing in vivo and in vitro models such as the LoC. Nonetheless, the LoC allows for molecular explorations of drug candidate effectiveness in liver‐specific cellular processes.

Previous studies unraveled the potential of LoC to study the effects of lanifibranor on MASLD‐associated cellular processes and demonstrated its potential to decrease lipid accumulation in hepatocytes.^[^ ^7^ ^]^ Yet here, we further increased the complexity of the LoC model by dissecting the roles of KCs and CICs. Indeed, it has been extensively demonstrated that immune cell accumulation and activation, either derived from the liver resident or from systemic circulation, are a hallmark of early and advanced MASLD.^[^ ^14^, ^30^, ^31^ ^]^ One may envision the relevance of this approach in alternative exploratory protocols, including the use of genetically modified animal primary cells, gene interference on specific LoC cell types, or targeted interventions on or depletion of CIC cell types. Additionally, the LoC may be part of more complex in vitro systems, by being connected to chips modeling other organs through serial perfusion networks, including but not limited to bone‐marrow‐ or adipose‐tissue‐on‐a‐chip.

MASLD is characterized by potent alterations of lipid metabolism. Resmetirom has been shown to promote body weight loss, as well as reduced triglycerides, apolipoprotein, and LDL‐cholesterol levels in patient serum in a phase 2 clinical trial.^[^ ^8^ ^]^ Furthermore, a newly‐released phase 3 clinical trial demonstrated that resmetirom was safe and well tolerated in adults with presumed MASH.^[^ ^32^ ^]^ In the LoC, we observed no changes in HDL‐ and LDL‐cholesterol levels, yet increased levels of triglycerides in the perfusate after resmetirom treatment. This observation was further associated with decreased lipid accumulation in hepatocytes in the LoC, and increased circulating levels of sphingomyelins and precursors, prompting us to assume that resmetirom effectively improved FFA metabolism in the LoC and export of FFA metabolites to the circulation, as reflected by increased levels of FFA metabolites in the perfusate. Increased ceramide levels are indicative of early MASLD and have been shown to promote hepatocyte death, while at later disease stages, increased ceramide levels would lead to increased S1P levels. Furthermore, increased FFA uptake has been shown to increase S1P levels. Furthermore, increased S1P was shown to promote liver fibrosis through HSC activation.^[^ ^24^ ^]^ Importantly, LoC and conventional hepatocyte metabolite analyses led to very different results. Those discrepancies may be explained by the presence of multiple cell types capable of sphingolipid metabolism and responding to sphingolipids, such as macrophages and LSECs.^[^ ^33^, ^34^ ^]^ This would demonstrate that the FFA metabolism can be further dissected in a culture system that includes multiple cell types. Thus, the LoC enabled the observation of complex metabolism‐related effects of resmetirom in vitro. This data further reflects the need for advancing MASLD in vitro models toward multicellularity with extensive flexibility in the type of data being generated from a defined set of experiments. These results also highlight the need for combining the LoC with other systems such as an adipose tissue‐on‐a‐chip, for further evaluating the impact of other organs in the clearance of circulating FFA‐metabolites. In line, APAP metabolism was reduced in the presence of CICs, revealing key, yet unexplored, mechanisms taking place in a multicellular system and requiring elaborate in vitro models for in‐depth analyses.

MASLD represents a global yet growing challenge to human well‐being and to our healthcare systems, mostly due to unhealthy lifestyles. While preventive measures should be the priority, the current situation requires finding therapeutic approaches for patients exhibiting varying degrees of this metabolic disorder leading to liver function impairment after a decade‐long MASLD progression. Intense efforts are thus deployed to decipher the pathomechanisms involved and find therapeutic candidates that could halt or reverse detrimental disease progression. Several clinical trials failed to demonstrate therapeutic effects, despite tremendous resource investments and promising preclinical data. One common issue with animal‐based models is inter‐species metabolism‐ and immune system‐related differences. Nevertheless, animal testing remains essential in pre‐clinical studies. European countries are regularly strengthening the regulations for conducting live animal‐based research due to ethical concerns and animal welfare. As a result, there is a growing interest in developing in vitro alternatives with increasing complexity.^[^ ^16^, ^35^ ^]^ In order to test the relevance of the LoC we isolated primary mouse cells from healthy mice (i.e., without any experimentation performed on the living animal). As for human material, it remains challenging to establish a reliable source of primary human cells of sufficient quality for research purposes. Ultimately though, facilitating human primary material use would accelerate the development of such methods, and open the path to personalized medicine.^[^ ^16^ ^]^ Here, despite their limitations, we used human cell lines combined with primary human blood cells, to evaluate the potential of the LoC to be used in human settings. Our data shows promising results prompting for the development of a fully primary human cell‐based LoC in the future. Several groups recently made important progress in the generation of (patient‐derived) iPSCs‐derived systems for modeling liver diseases. While these methods present important advantages to overcome primary cell shortage, they remain technically challenging and rely on the use of complex and resource‐demanding culture conditions that bring novel burdens on liver research. Nonetheless, we expect iPSC‐derived liver disease modeling to drastically improve in the near future.

Overall, the LoC represents an attractive and promising approach for the characterization of pathology‐driving mechanisms and pre‐screening of candidate drug compounds or molecular pathways, prior to using living animal models for more complex, organism‐based validations. To our knowledge, the LoC as herein described represents the first model to offer means to dynamically assess cellular status and functions, either directly by imaging methods on the biochip or by performing flow cytometry on circulating cells, or by measuring circulating markers of hepatocyte injury, also reflecting the engagement of inflammatory processes and metabolites. Indeed, we optimized the read‐out possibilities so that singular experiments can be maximized and lead to the generation of extensive, biologically‐relevant findings. The LoC may thus participate in the global efforts to increase the pace of drug discovery and reduce research animal use while highlighting the values of maintaining in vivo models prior to human testing and clinical applications.

## 4 Experimental Section

### Animals

C57BL/6J wild‐type mice (18–24‐week‐old) and fluorescent reporter transgenic (actin‐dsRed and actin‐CFP in a B6 genetic background) mice were killed by isoflurane overdose prior to any experimentation and used as a primary cell source in this study. Archival formalin‐fixed paraffin‐embedded liver tissues in this study were harvested from animal models, which were administrated with choline‐deficient, *L*‐amino acid‐defined, high‐fat diet (CDAHFD) for 6 weeks, following by the administration of lanifibranor and CDAHFD (30 mg k^−1 ^g day^−1^) for 2 weeks ^[^ ^7^ ^]^ or injected once with acetaminophen (APAP, 250 mg k^−1 ^g day^−1^) and mice were killed and organs were collected after 24 h.^[^ ^36^ ^]^ Isolation procedures are detailed in SI (Methods Section). All animal procedures were approved by Lageso Berlin, Germany (Approval numbers: G0243‐19 and TCH‐020‐22).

### Liver‐On‐A‐Chip Preparation

Primary liver and blood cells were isolated from healthy adult mice. Briefly, the liver was perfused with a collagenase solution and the cells in suspension were further separated by gradient and magnetic bead‐based methods. Primary liver and blood cell isolation and seeding protocols are explained in more details in the Supporting Information.

### Cytokine and Transaminase Measurements

The LoC perfusate or cell culture supernatant were collected and centrifuged at 1000 × g for 10 min at 4 °C. The resulting supernatant samples were stored at −80 °C until analysis. Aspartate aminotransferase (AST) and alanine transaminase (ALT)levels were measured by Labor Berlin–Charité Vivantes GmbH, Berlin, Germany, using standard procedures. Cytokine levels were measured using the LEGENDplex Mouse inflammation panel (Biolegend, USA) according to the manufacturer's manuals (Table S1, Supporting Information). BD FACSCanto II set up in the PE and APC channels was used to perform the measurements according to the manufacturer's manuals. Due to the out‐of‐range measurements despite statistically different values between the groups, cytokine values were depicted in arbitrary units calculated as the estimated relative concentrations after standard curve extrapolation.

### Flow Cytometry for Circulating Immune Cell (Cic) Characterization

Mouse CICs were isolated from CFP mice. After red blood cell lysis, CICs were perfused in the LoC for 30 min, then the perfusate was collected and cells were centrifuged at 400 × g for 5 min. The cells were then incubated with a fixable viability dye (Zombie NIR Fixable Viability Kit; Biolegend, USA) at a 1:5000 dilution for 10 min at 4 °C, followed by incubation with fluorochrome‐conjugated antibodies (Table S1, Supporting Information) in blocking buffer (PBS + 2% BSA + 2% normal mouse/rat/rabbit/human serum) for 20 min at 4 °C. Cells were fixed with PBS containing 1% formalin for 10 min at 4 °C. Finally, the cells were resuspended in 200 µL PBS and 10 µL counting beads (10^6^ beads mL^−1^) were added to each sample. Multispectral flow cytometry was performed using the Cytek Aurora.

### Gene Expression Analysis

Total RNA was extracted from the cells attached to the LoC membrane by using the RNeasy Kit (Qiagen, Germany) according to the manufacturer's instructions. Quantitative real‐time PCR was carried out using the Applied Biosystems Real‐Time PCR System (ThermoFisher, USA) and SYBR RT‐PCR kits (Roche, Switzerland). The −ΔΔCycle threshold (Ct) analysis method was used to assess relative mRNA expression normalized to *Actb*. Primer sequences are listed in Table S2 (Supporting Information).

### Multiplex Immunohistochemistry

Sequential multiplex immunostaining was performed as previously described.^[^ ^30^, ^37^, ^38^ ^]^ The antibody elution buffer was prepared by mixing 675 µL distilled water, 125 µL 0.5 m Tris‐HCl pH 6.8, 200 µL 10% (w/v) sodium dodecyl sulfate, and 8 µL 2‐mercaptoethanol. The list of antibodies is provided in Table S1 (Supporting Information).

### Sphingolipid Measurement

Perfusates were subjected to lipid extraction using 1.5 mL methanol/chloroform (2:1, v:v) containing internal standards. Chromatographic separation was achieved on a 1290 Infinity II HPLC (Agilent Technologies, Waldbronn, Germany) equipped with a Poroshell 120 EC‐C8 column (3.0 × 150 mm, 2.7 µm; Agilent Technologies) guarded by a pre‐column (3.0 × 5 mm, 2.7 µm) of identical material. Details are provided in SI (Methods Section). MS/MS analyses were carried out using a 6495C triple‐quadrupole mass spectrometer (Agilent Technologies) operating in the positive electrospray ionization mode (ESI+).^[^ ^39^ ^]^ Quantification was performed with MassHunter Quantitative Analysis software (version 10.1, Agilent Technologies).

### Statistical Analysis

The GraphPad Prism 9.0 software (GraphPad Software, USA) and R studio (version: 2023.03.1 Build 446; plugins “ggplot2”) were used to generate plots.^[^ ^40^ ^]^ Heatmaps displayed in most figures represent relative values as compared to the appropriate control. Single data points are shown in the Supporting Information Figures. The statistical tests used in this study are indicated in the figure legend for each panel. Data were presented as the mean ± S.D. ‘*p* < 0.05′ was considered to be significantly different.

All animal procedures were approved by Lageso Berlin, Germany (Approval numbers: G0243‐19 and TCH‐020‐22). The study with hLoC and the isolation and use of PBMCs and HUVECs was approved by the ethics committee of the Jena University Hospital (2020–1684, 3939–12/13). All donors were informed about the aim of the study and gave written consent.

## Conflict of Interest

A.S.M. holds equity in and consults for Dynamic42 GmbH. FT's lab has received research funding from Allergan, Bristol‐Myers Squibb, Gilead and Inventiva. FT has received honoraria for consulting or lectures from Astra Zeneca, Gilead, AbbVie, BMS, Boehringer, Madrigal, MSD, GSK, Intercept, Falk, Ionis, Inventiva, Merz, Pfizer, Alnylam, NGM, CSL Behring, Novo Nordisk, Novartis. The other authors declare no conflict of interest.

## Author Contributions

H.L. and A.G. conceptualized the study and developed methodology, and performed the investigation with G.Y., M.S.K., F.S., J.H., and M.H. Data was analyzed by H.L., G.Y., M.S.K., F.S., F.H., T.P., B.K., A.M., F.T., and A.G. The manuscript was written by H.L. and A.G., reviewed by F.S., B.K., A.M., and F.T. and approved by all authors. F.T. and A.G. acquired funding. A.G. supervised the research.

## Supporting information

Supporting Information

## Data Availability

The data that support the findings of this study are available from the corresponding author upon reasonable request.
