# Supplementary material for: Dissecting Acute Drug‐Induced Hepatotoxicity and Therapeutic Responses of Steatotic Liver Disease Using Primary Mouse Liver and Blood Cells in a Liver‐On‐A‐Chip Model
Source: Adv Sci (Weinh). 2024 Jun 13;11(30):2403516. doi: 10.1002/advs.202403516 (PMC11321671; doi:10.1002/advs.202403516)
Supplement: Supplementary file 1 — Supporting Information [file ADVS-11-2403516-s001.pdf]

ADVANCED SCIENCE
Open Access

Supporting Information

for *Adv. Sci.*, DOI 10.1002/advs.202403516

Dissecting Acute Drug-Induced Hepatotoxicity and Therapeutic Responses of Steatotic Liver Disease Using Primary Mouse Liver and Blood Cells in a Liver-On-A-Chip Model

*Hanyang Liu, Guo Yin, Marlene Sophia Kohlhepp, Fabian Schumacher, Jana Hundertmark, Mohamed I. Abdelwahab Hassan, Felix Heymann, Tobias Puengel, Burkhard Kleuser, Alexander Sandy Mosig, Frank Tacke and Adrien Guillot**

Supporting Information

Title

Dissecting pathomechanisms and therapeutic responses of steatotic liver disease using primary mouse liver and blood cells in a liver-on-a-chip model

Hanyang Liu[1], Guo Yin[1], Marlene Sophia Kohlhepp[1], Fabian Schumacher[2], Jana Hundertmark[1], Mohamed I Abdelwahab Hassan [3], Felix Heymann[1], Tobias Puengel[1], Burkhard Kleuser[2], Alexander S. Mosig[3], Frank Tacke[1], Adrien Guillot[1,]*

1. Charité Universitätsmedizin Berlin, Campus Virchow-Klinikum and Campus Charité Mitte, Department of Hepatology & Gastroenterology, Berlin, Germany
2. Freie Universität Berlin, Institute of Pharmacy, Königin-Luise-Str. 2+4, Berlin, Germany
3. Institute of Biochemistry II, Center for Sepsis Control and Care, Jena University Hospital, Germany

1. Supplementary materials and methods

1.1. Materials for liver and blood primary cell isolation and culture

1.1.1. Enzymes/chemicals

1) Protease (Sigma-Aldrich, cat. no. P5147)
2) Collagenase type 4 (CellSystems, cat. no. LS004186)
3) DNase I (Roche, cat. no. 10 104 159 001)
4) Nycodenz (Accurate Chemical, cat. no. 1002424)
5) Sodium chloride (NaCl; Sigma-Aldrich, cat. no. S3014)
6) Potassium chloride (KCl; Sigma-Aldrich, cat. no. P9333)
7) Sodium Phosphate monobasic monohydrate ($NaH_2PO_4 \cdot H_2O$; Fisher Scientific, cat. no. S369)
8) Sodium phosphate (Na_2HPO_4; Sigma-Aldrich, cat. no. S9763)
9) HEPES (Sigma-Aldrich, cat. no. H4034)
10) Sodium bicarbonate ($NaHCO_3$; Sigma-Aldrich, cat. no. S5761)
11) EGTA (Sigma-Aldrich, cat. no. E4378)
12) D-(+)-Glucose (Sigma-Aldrich, cat. no. G8270)
13) Calcium chloride dihydrate ($CaCl_2 \cdot 2H_2O$; Sigma-Aldrich, cat. no. C7902)
14) Magnesium chloride hexahydrate ($MgCl_2 \cdot 6H_2O$; Sigma-Aldrich, cat. no. 930970)
15) Magnesium sulfate heptahydrate ($MgSO_4 \cdot 7H_2O$; Sigma-Aldrich, cat. no. 230391)
16) Potassium phosphate monobasic (KH_2PO_4; Sigma-Aldrich, cat. no. 60229)
17) Albumin from bovine serum, BSA (Sigma-Aldrich, cat. no. A9430)
18) Paracetamol (Sigma-Aldrich, cat. no. BP371)
19) Palmitic acid, PA (Sigma-Aldrich, cat. no. P0500)
20) Oleic acid, OA (Sigma-Aldrich, cat. no. O1008)
21) Lanifibranor (Biozol, cat. no. 927961-18-0)
22) Resmetirom (Biozol, cat. no. 10033494)
23) Paraformaldehyde, PFA ($HO(CH_2O)_nH$, Sigma-Aldrich, cat. no. 158127)
24)

1.1.2. Solutions

1) Dulbecco's phosphate-buffered saline, DPBS (Gibco, cat. no. 14190144)
2) Phosphate-buffered saline, PBS (Gibco, cat. no. 70011044)
3) Hanks' Balanced Salt Solution, HBSS, Calcium, Magnesium (Gibco, cat. no. 24020117)
4) William’s E medium (Gibco, cat. no. W1878-500ML)
5) Fetal bovine serum, FBS (Gibco, cat. no. A4736201)
6) Normal Goat Serum, NGS (10%) (ThermoFisher, cat. no. 50062Z)
7) Primary hepatocyte maintenance supplements (Gibco, cat. no. CM4000)
8) Lysing buffer (10X) (BioLegend, cat. no. 420301)
9) Collagen I, rat tail (ThermoFisher, cat. no. A1048301)
10) Acetic acid solution (CH_3CO_2H, Sigma-Aldrich, cat. no. 45754)
11) Percoll-100% (Sigma-Aldrich, cat. no. P1644)
12) Triton X-100 solution (Sigma-Aldrich, cat. no. 93443)
13) Isoflurane (CP-pharma, cat. no. 798-932)
14) Ethanol solution 70 % (ThermoFisher, cat. no. 15542393)
15) BD Pharm Lyse™ Lysing Buffer (10X) (BD, cat. no. 555899)
16) EGTA buffer: Prepare the solution by dissolving the components of the recipe given below in 1 L of ddH_2O. Adjust the pH to 7.4 and filter the solution through a 0.2-µm bottle-top filter. The constituted solution can be stored at 4°C for up to 6 months.

**Reagent**	**Final concentration (mg/liter)**
NaCl	8,000
KCl	400
NaH2PO4·H2O	88.17
Na2HPO4	120.45
HEPES	2,380
NaHCO3	350
EGTA	190
D-(+)-Glucose	900

17) Enzyme buffer: Prepare the solution by dissolving the components of the recipe given below in 1 L of ddH_2O. Adjust the pH to 7.4 and filter the solution through a 0.2-µm bottle-top filter. The ready-to-use solution can be stored at 4°C for up to 6 months.

**Reagent**	**Final concentration (mg/liter)**
NaCl	8,000
KCl	400
NaH2PO4·H2O	88.17
Na2HPO4	120.45
HEPES	2,380
NaHCO3	350
CaCl2·2H2O	560

18) GBSS/A buffer: Prepare the solution by dissolving the components of the recipe given below in 1 L of ddH_2O. Adjust the pH to 7.4 and filter the solution through a 0.2-µm bottle-top filter. The constituted solution can be stored at 4°C for up to 6 months.

**Reagent**	**Final concentration**

	(mg/liter)
KCl	370
MgCl2·6H2O	210
MgSO4·7H2O	70
Na2HPO4	59.6
KH2PO4	30
Glucose	991
NaHCO3	227
CaCl2·2H2O	225

19) GBSS/B buffer: Add 8 g/L NaCl into 1 L of GBSS/A buffer described above to make GBSS/B buffer. Adjust the pH to 7.35 – 7.4 and filter the solution through a 0.2-µm bottle-top filter. Constituted solution can be stored at 4°C for up to 6 months.
20) Digestion buffers: Prepare the solution by dissolving the components of the recipe given below. Buffers need to be freshly made for each experiment. Filter the buffer with a 0.22-µm filter before use.
    a) Digestion buffer I: Dissolve 4.4U collagenase type 4 in 50 mL enzyme buffer.
    b) Digestion buffer II: Dissolve 4.4U collagenase type 4, 40 µg DNase I and 4.5 mg pronase in 50 mL enzyme buffer.
21) DNase I solution: Dissolve 100 mg in 100 mL PBS to make 1 mg/mL solution.
22) Percoll-50% solution: Dissolve 10.8 mL Percoll-100% and 1.2 mL 10X-PBS in 14.5 mL 1X-PBS to make Percoll-50% solution. Mix it thoroughly.
23) Nycodenz solution: Dissolve 4.94 g nycodenz in 15ml GBSS/A buffer and filter it through a 0.22-µm filter. Adjust the solution to 17ml. Optional: Add phenol red to indicate gradient layers.
24) Magnetic-activated cell sorting (MACS) buffer: Dissolve 250 µg BSA and 37.2 µg EDTA in 50 mL DPBS. Filter it through a 0.22-µm filter. The constituted solution can be stored at 4°C for up to 2 weeks.
25) Collagen-coating buffer: Dissolve 660 µL collagen-I in 50 mL DPBS containing 0.12% acetic acid. Filter it through a 0.22-µm filter.
26) Blocking buffer: 5% normal goat serum and 0.3% Triton-X in DPBS.
27) Antibody buffer: 1% BSA and 0.3% Triton-X in DPBS.
28) Free fatty acid (FFA) solution: Dissolve PA or OA in DPBS containing 1% BSA, Ratios of PA and OA in FFA mixture can be adjusted for different perspectives. Final working FFA concentration is 30 mM.
29) Blocking buffer: Respectively dilute NGS and TritonX-100 into the DPBS to make 5% NGS and 0.3% TritonX-100 as final concentrations.
30) Antibody buffer: Respectively dilute BSA and TritonX-100 into the DPBS to make 1% BSA and 0.3% TritonX-100 as final concentrations.
31) VectaMount AQ Aqueous Mounting Medium (Vectorlabs, cat. no. H-5501)

1.1.3. Equipments

1) Microfluidic biochips were made from polybutylene terephthalate (PBT) and obtained from Dynamic42 GmbH (Jena, Germany). The pore diameter in the membrane separating the two chambers is 8 micrometer. The design and technical parameters have been previously described [1, 2].
2) Perfusion pump and tubing (Ismatec REGLO digital, MS-CA-4/12–100).
3) I.V catheter (JELCO 22G IV catheter)

4) Needle (0.9 X 40 mm BL/LB, Sterican)
5) Blood vessel clamp and scissors (Fine Science Tools, cat. no. 18039-45)
6) Bottle-top filter, 0.2 µm 150 mL (Corning, cat. no. 431161)
7) Shakable water bath
8) Syringes: 3 and 20 ml
9) Sterile filter for syringe, 0.22 µm (Millipore, cat. no. SLGP033RB)
10) 70-um strainer (Corning, cat. no. 352350)
11) Sterile pipettes: 5, 10 and 25 mL (ThermoFisher, cat. no. 170366N, 170356N and 170357N)
12) Falcon tubes, 15 mL (Corning, cat. no. 352095)
13) Falcon tubes, 50 mL (Corning, cat. no. 352070)
14) Sterile Petri dishes, 100 mm (Corning, cat. no. 70165-100)
15) MS columns (Miltenyi, cat. no. 130-042-201)
16) QuadroMACS Separator (Miltenyi, cat. no. 130-091-051)
17) AccuSpin 1R Centrifuge: Refrigerated (ThermoFisher, cat. no. 4168)
18) Mouse operation area (Styrofoam board, handmade)
19) InkJet Plus Microscope Slides (ThermoFisher, cat. no. 12-550-109)

1.1.4. Antibodies, dyes and microbeads

All antibodies, dyes and microbeads that were used for fluorescence detection and cell sorting are listed in Table S2.

1.2. Methods

1.2.1. All-from-one mouse primary liver cell isolation (see the isolation procedures in Fig. 1A)

1.2.1.1. Mouse liver perfusion and digestion

1) Before starting, pre-warm EGTA / enzyme buffers in the 42°C water bath; assemble tubes and catheters in the pump.
   NOTE: The temperature can be adjusted to ensure solutions perfused in mouse liver at mouse body temperature.
2) Sacrifice mice with the inhalation of isoflurane (60 µg/g weight) for 3 min.
3) Fix mouse feet on the operating board, with the abdominal side up. Spray the abdomen with 70% ethanol to sterilize the area and wet the fur.
4) Open the abdominal cavity. Gently push intestines and colon to the right side, to expose the portal vein and inferior vena cava without punching into the organs.
5) Cannulate the portal vein using an I.V catheter. Perfuse the liver with EGTA buffer (40 mL) at a flow rate of 2 mL/min. Section the vena cava at a distal position to allow for perfusate clearance.
   OPTIONAL: For blood collection, insert a needle into inferior vena cava prior to starting perfusion. Start perfusion and collect blood with a 1.5 mL Eppendorf tube. Pull out the needle after collection.
6) **CAUTION**: It is critical to avoid bubbles during perfusion. Check for the presence of bubbles in the tubing prior to inserting the catheter. When switching perfusion reagents, make sure to stop the pump prior to transferring the tubing from one tube to the other.
   NOTE: The liver should appear homogeneously brighter within seconds after initiating the perfusion and cutting the inferior vena cava. If this is not the case, the perfusion is not optimal, and this will lead to difficulties during cell isolation.

NOTE: During the perfusion, it is necessary to press inferior vena cava with a vessel lamp for 30-40 sec to check the perfusion quality. Liver lobes get inflating rapidly.

7) Continue perfusion with the digestion buffer I (50 mL) at a flow rate of 2 mL/min.
8) Stop the perfusion and remove the catheter. Carefully remove the whole liver (dissect and discard the gallbladder).
9) Gently smash the liver tissue by vessel lamps for further isolation procedures..
OPTIONAL: Separate liver tissues if the multiple cell type isolation is required. For the HSC, KC and LSEC isolation, an extra digestion (in a 37℃ incubator with the mild shaking level for 25 - 35 min) is necessary.

1.2.1.2.Mouse primary hepatocyte isolation

1) Pass the liver cell suspension through a 70-µm strainer to a 50 mL Falcon tube. Fill up the tube with HBSS to 50 mL. Centrifuge the cell suspension at 4°C, 50 g for 5 min.
2) Aspirate the supernatant and resuspend the hepatocyte pellet in 50 mL HBSS. Centrifuge again the cell suspension at 4°C, 50 g for 5 min.
CAUTION: Keep the supernatant for MACS procedures if necessary.
3) Aspirate the supernatant and resuspend the hepatocyte pellet in 50 mL Percoll-50% solution. Centrifuge cells at 4°C, 400 g for 10 min.
4) Aspirate the supernatant and resuspend the hepatocyte pellet with 50 mL HBSS buffer. Centrifuge the hepatocytes at room temperature, 50 g for 10 min.
5) Resuspend the hepatocyte pellet in medium for counting and seeding.

1.2.1.3.Mouse primary hepatic stellate cell isolation

1) Filter the liver cell suspension through a 70-um strainer. Fill up the tube with GBSS/B buffer to 50ml. Centrifuge the cell suspension at 4°C, 580 g for 10 min.
2) Aspirate the supernatant until 10 mL remains in the tube. Add 100 µL of DNase I solution and fill it up to 50 mL with GBSS/B buffer. Centrifuge again at 580g for 10 min at 4 °C.
3) Aspirate the supernatant until 10 mL remains in the tube. Add 100 µL of DNase I solution and fill it up to 32 mL with GBSS/B buffer. Add 16 mL Nycodenz solution and mix thoroughly. Split 48 mL cell-Nycodenz suspension equally into four 15-ml Falcon tubes.
4) Gently overlay with cell-Nycodenz suspension with 1.5 mL of GBSS/B buffer using a 3-ml syringe with a 26-gauge needle attached. Centrifuge the suspension at 4 °C, 1,380g with no brake for 17 min.
CAUTION: Make sure to overlay the GBSS/B buffer gently above the cell-Nycodenz suspension to create a discontinuous gradient. A clear separation should be observed.
5) Use a 5-ml pipette to collect the cells and transfer them into a new 50-ml Falcon tube. Resuspend the cell pellets by MACS buffer for the magnetic cell sorting.
OPTIONAL: Repeat the procedure (3 – 5) with cell pellets to obtain more cells.
6) Fill up the Falcon tube to 50 mL with GBSS/B buffer. Gently resuspend the collected HSCs. Centrifuge at 4 °C, 580g for 10 min.
7) Resuspend the cell pellet in medium for counting and seeding.

1.2.1.4.Magnetic cell sorting (for KCs and LSECs)

1) Centrifuge cell suspension at 4°C, 400 g for 10 min.
2) Discard supernatant and resuspend in 90 µL MACS buffer per 10^7 cells.
3) Respectively add 10 µL /10^7 cells magnetic beads (Anti-F4/80 for KCs or Anti-CD146 for LSECs) into the cell suspension. Mix well and incubate on ice for 15 min.

4) Add 900 µL MACS buffer per 10^7 cells and centrifuge at 4°C, 300 g for 10 min.
5) Discard the supernatant and resuspend cells in 500 µL of MACS buffer. Place the MS column in the magnetic separator and rinse it with 500 µL of MACS buffer. Add the cell suspension into the column. Collect the flow-through containing unlabeled cells for a next magnetic cell sorting.
   OPTIONAL: LS column can be used if higher amount of cell sorting is required.
6) Wash column with 3x 500 µL of MACS buffer. Move the column out from the magnetic separator. Add 1 mL MACS buffer into the column. Firmly flush out the buffer from the column by the plugger. Collect the labelled retained cells by a 15-mL Falcon tube. Centrifuge the cell suspension at 4°C, 400 g for 10 min.
7) Resuspend the cell pellet in medium for counting and seeding.

1.2.1.5. Mouse Peripheral Blood Mononuclear Cell (circulating immune cells, or CIC) isolation

NOTE: This section refers to blood collected from the vena cava during the liver perfusion. If needed, more blood may be collected from the heart using a 1 mL syringe containing 0.5 mL EGTA buffer before or during the liver perfusion.

1) Centrifuge blood samples at 4°C, 400 g for 10 min.
2) Aspirate supernatant and resuspend cell pellets with 1 mL 1X red blood cell lysing buffer.
3) Incubate cell suspension on ice for 15 min.
4) Centrifuge at 4°C, 400 g for 10 min.
5) Aspirate supernatant and resuspend the cell pellet in cell culture medium for counting and seeding.

1.2.2. LoC model assembly and disease modeling (see the biochip structure in Fig. 2B)

1) Sterilize the surface and inner cavities of biochip by pipetting in 70% ethanol and incubating for 40 min.
2) Wash cavities 3 times with DPBS buffer.
3) Pipet 500 mL collagen coating buffer in each cavity and incubate for 15 min.
4) Wash cavities 3 times by DPBS buffer. Fill 500 µL medium in each cavity. Block connections between cavities by plugs.
5) Cell seeding and intervention strategies:
   a) On Day 1, seed 300,000 LSECs and 100,000 liver macrophages in the lower cavity. Close lids and flip down the chip. Incubate overnight at 37°C, 5% CO_2.
   b) On Day 2, flip back the chip. Seed 300,000 hepatocytes and 100,000 HSCs in the upper cavity. Close lids. Incubate at 37°C, 5% CO_2 for overnight.
   c) On Day 3, add FFA treatment (300 µM, PA:OA = 1:1), FFA + 20 µM lanifibranor, 25 mM APAP or vehicle (0.1 mM BSA for FFAs or 1% PBS for APAP). Incubate at 37°C, 5% CO_2 overnight.
6) Bio-chip perfusion strategies:
   a) Connect the tubing to each lower cavity. Add 1 mL medium containing 100,000 CICs into each reservoir. Include FFA treatment as described above (300 µM, PA:OA = 1:1), FFA + 20 µM lanifibranor, 25 mM APAP or vehicle (0.1 mM BSA for FFAs or 1% PBS for APAP) in each reservoir.
   b) Start perfusion in separated pumping channels at 50 µL / min.
   c) Transfer chip-pump assemblies into the incubator. Incubate at 37°C, 5% CO_2 for 30 min – 48 hours depending on the experimental needs.

CAUTION: Bubbles should be avoided at all time during cell seeding into the biochip, medium changing and CIC perfusion.

7) Sampling strategies:
   a) Apply live cell dye on cells and observe under microscope.
   b) Harvest the perfusion medium and centrifuge at 4°C, 400g for 10 min. Collect CICs in the cell pellets. Keep the supernatant for further analyses (e.g. transaminases, cytokine levels, see corresponding sections).
   c) Cut off membranes to harvest resident cells for cell visualization or RNA extraction.

1.2.3. Fluorescence detection

1) Live cell staining and detection
   Hoechst 33342 is used to stain cell nucleus; the CellMask plasma membrane stain kit is used to indicate cell membrane and trace cells; the Apoptosis/ Necrosis Assay Kit is used to determine apoptotic, necrotic and viable cells; the BODIPY 505/515 is used to label intracellular lipid droplets. Experiments were all performed following manufacturers' manuals.
2) Immunocytochemistry (ICC)
   a) Fix the cells (in the chip or on the collected membranes) with 4% PFA buffer at room temperature for 15 min.
      OPTIONAL: Cells labelled by live cell dyes can be processed to ICC approaches after fixation. Permeabilize cells and block unspecific membrane antigens by the blocking buffer at the room temperature for 30 – 60 min.
   b) Freshly dilute primary antibodies into the antibody buffer following manufacturers' manuals. Apply antibody solutions on cells and incubate at 4℃ overnight or at room temperature for 4 h.
   c) Wash cells twice with DPBS.
   d) Freshly dilute secondary antibodies into the antibody buffer following manufacturers' instructions. Apply antibody solutions on cells and incubate at room temperature for 30 min.
   e) Wash cells twice with DPBS.
   f) Dissolve DAPI into DPBS to 0.1 µg/mL. Apply DAPI solution on cells and incubate at room temperature for 10 min.
   g) Wash cells twice by DPBS.
   h) Observe cells under the fluorescence microscope.

OPTIONAL: If necessary, mount cells by the VectaMount AQ aqueous mounting medium and cover cells with glass slides. Immersed into DPBS, glass slides can be gently detached in 30 min. Therefore, cells or membranes can be recycled for IF staining or other perspectives.

1.2.4. Human cell materials and human liver-on-a-chip methods

1.2.4.1. Cell culture:

Human umbilical vein endothelial cells (HUVECs) were isolated from human umbilical cord veins 3 × 10^5/cm^2 HUVEC cells in endothelial cell growth medium MV (Promocell, C-22020) supplemented with 1% penicillin-streptomycin (Invitrogen, 15140-122). Donors were informed about the aim of the study and gave written consent. HUVECs were used until passage three.
Monocytes were isolated from human blood. Briefly, peripheral blood mononuclear cells (PBMCs) were isolated by Ficoll density gradient centrifugation and seeded at a density of 1

$\times 10^6$ /cm^2 cell in x-vivo 15 (Lonza, 02-060F) supplemented with 10 ng/mL GM-CSF (Peprotech, 300-30-100UG), 10 ng/mL M-CSF (Peprotech, 300-25-100UG), and 1% penicillin-streptomycin (Invitrogen, 15140-122). After two hours of incubation at 5% CO_2 and 37 °C, the cells were washed twice with X–VIVO 15 medium (Lonza, 02-060F). Adherent monocytes were then cultivated for in X-VIVO 15 supplemented with 10ng/mL GM-CSF (Peprotech, 300-30-100UG), 10 ng/mL M-CSF (Peprotech, 300-25-100UG), and 1% penicillin-streptomycin (Invitrogen, 15140-122).
HepaRG cells were obtained from Biopredic International (Rennes, France). Cells were seeded at a density of 2.7×10^4 cells/cm^2 cells and cultured in William's E media (1x) [2,2g/L $NaHCO_3$] (Gibco, 22551-022) supplemented with fetal bovine serum (FBS) (Gibco, 10270-106), 5 mg/mL insulin (Sigma Aldrich, 11061-68-0), 50 µM hydrocortisone-hemisuccinate (Merck, H2270-100mg), 2% DMSO (Sigma, D2650-100ml), and 100 U/mL penicillin / 100 mg/mL streptomycin mixture (Pen/Strep) (Invitrogen, 15140-122).
Human hepatic stellate cell line (LX-2) is generous given by Ralf Weiskirchen (Institute of Molecular Pathobiochemistry, University Hospital Aachen) and were cultivated 3×10^5/cm^2 LX-2 cells in DMEM with high glucose containing GlutaMAX™ supplement and pyruvate (Gibco, 31966-02) media supplemented with 10% FBS (Gibco, 10270-106), and 1% penicillin-streptomycin (Invitrogen, 15140-122). The cells were cultured in a humidified cell incubator at 5% CO_2 and 37 °C.

1.2.4.2.Human Liver-on-chip (hLoC)
3×10^5 HUVECs cells were seeded with 1×10^5 macrophages at the same day in endothelial cell growth medium MV (Promocell, C-22020) supplemented with 1% penicillin-streptomycin (Invitrogen, 15140-122), 10ng/mL GM-CSF (Peprotech, 300-30-100UG), 10 ng/mL M-CSF (Peprotech, 300-25-100UG) at the lower cavity. The chip was flipped down and incubated overnight in a cell culture incubator with 5% CO2 at 37 °C.
At the following day, 2×10^5 HepaRG and 1×10^5 LX-2 cells were mixed in William's E media (1x) [2,2g/L $NaHCO_3$] (Gibco, 22551-022) supplemented with FBS (Gibco, 10270-106), 5 mg/mL insulin (Sigma Aldrich, 11061-68-0), 50 µM hydrocortisone-hemisuccinate (Merck, H2270-100mg), 2% DMSO (Sigma, D2650-100ml), and 100 U/mL penicillin / 100 mg/mL streptomycin mixture (Pen/Strep) (Invitrogen, 15140-122). Cells were seeded on the opposite side of the chip membrane and cultured in a cell culture incubator with 5% CO2 at 37 °C for 24 hours.
At the third day, treatments with free fatty acids (FFA) [300 µM, mixture of palmitic acid (PA) (Sigma-Aldrich, P0500) and oleic acid (OA), v/v = 1:1] (Sigma-Aldrich, O1008), FFA mixture supplemented with 20µM lanifibranor (Biozol, 927961-18-0) and 10% BSA (Sigma-Aldrich, A9430) was performed by perfusion of the hLoC.

1.2.4.3.Biochip perfusion
hLoC was perfused on both sides for 24 hours with a flow rate of 50 µL / min using a peristaltic pump (Cole-Parmer™, Ismatec™ Reglo ICC Digital). 1 mL medium containing 100,000 CICs (total PBMCs) was circulated at the lower cavity containing HUVEC / macrophages. Medium containing FFAs, FFAs + 20 µM lanifibranor, and 10% BSA was added to the medium perfusing both sides of the membrane. After perfusing chips with CICs for 30 minutes, flow cytometry analysis of the flow through medium containing nonadherent CICs was performed. Lipid accumulation was measured at 24h after starting FFA treatment.

1.2.4.4.Staining of CICs and flow cytometry
Cells were collected from perfusate by centrifugation and stained for viability using Zombie UV™ Fixable Viability Kit (BioLegend, 423107) for 10 minutes at 4℃. Cells were

subsequently stained with antibodies provided in Table S1, fixed with 4% PFA in PBS for 10 minutes at 4℃ and cells analyzed using a BD LSR Fortessa flow cytometer (Becton Dickinson).

1.2.4.5.BODIPY 505/515 lipid staining and Hoechst DNA staining:

BODIPY solution (Thermo Fisher, D3921) and Hoechst (Sigma Aldrich, H3342) were diluted in PBS 1:1000 and incubated with live cells in the dark for 15 min at 37 °C. After washing twice with PBS, cells were analyzed by immunofluorescence microscopy using an AxioObserver Z1 microscope equipped with Apotome2 (Carl Zeiss AG, Jena, Germany)

1.2.4.6.Cytokine measurement

Cytokine concentrations were measured using multiplex bead-based immunoassays (LEGENDplex, BioLegend, 740808) on a flow cytometer BD LSR Fortessa (Becton Dickinson). Data analysis was performed using manufacturers data analysis software (BioLegend, SanDiego, CA, USA).

1.2.4.7.Ethics

The study with hLoC and the isolation and use of PBMCs and HUVECs was approved by the ethics committee of the Jena University Hospital (2020–1684, 3939–12/13). All donors were informed about the aim of the study and gave written consent.

1.2.5. Analysis of APAP metabolization by HPLC coupled to high-resolution mass spectrometry (HRMS)

LoC perfusates and 2D cell culture supernatants were centrifuged at 10,000 g for 10 min and then subjected to HPLC-HRMS analysis. Chromatographic separation was achieved on a 1290 Infinity II HPLC (Agilent Technologies) equipped with a Zorbax Eclipse Plus C18 column (2.1 x 50 mm, 1.8 µm; Agilent Technologies). A mobile phase system consisting of water and acetonitrile, both acidified with 0.1% formic acid, was used for gradient elution at a flow rate of 0.35 mL/min. The HPLC eluate was directed to an electrospray ionization interface operated in positive mode (ESI+). Ions were analyzed using a 6550 iFunnel quadrupole time-of-flight (QTOF) mass spectrometer (Agilent Technologies). QTOF analysis was performed in scan mode in the range of *m/z* 50 to 1,700 with a scan rate of 3 spectra/s. The reference masses *m/z* 121.0509 and *m/z* 922.0098 were continuously supplied by an isocratic pump. The identity of the APAP metabolites was confirmed by the mass error ($\Delta m/z$) of less than 3 ppm and the isotopic pattern, both determined using MassHunter Qualitative Analysis software (version 10.0, Agilent Technologies).

1.2.6. Sphingolipid quantification in liver-on-a-chip perfusates by HPLC-ESI-MS/MS

Perfusates (300 µL) were subjected to lipid extraction using 1.5 mL methanol/chloroform (2:1, v:v) containing the internal standards d_7-dihydrosphingosine (d_7-dhSph), d_7-sphingosine (d_7-Sph), d_7-sphingosine 1-phosphate (d_7-S1P), 17:0 ceramide (d18:1/17:0; 17:0 Cer), d_{31}-16:0 sphingomyelin (d18:1/16:0-d_{31}; 16:0-d_{31} SM), 17:0 glucosyl(β) ceramide (d18:1/17:0; 17:0 HexCer) and 17:0 lactosyl(β) ceramide (d18:1/17:0; 17:0 LacCer) (all Avanti Polar Lipids, Alabaster, AL, USA). Extraction was facilitated by incubation at 48°C with gentle shaking (120 rpm) overnight. To reduce interference from glycerolipids, samples were saponified with 150 µL 1 M methanolic KOH for 2 h at 37°C with gentle shaking (120 rpm) followed by neutralization with 12 µL glacial acetic acid. After centrifugation at 2,200 g for 10 min at 4°C, organic supernatants were evaporated to dryness using a Savant SpeedVac concentrator (Thermo Fisher Scientific, Dreieich, Germany). Dried residues were reconstituted in 200 µL acetonitrile/methanol/water (47.5:47.5:5 (v:v:v), 0.1% formic acid) and subjected to HPLC-

ESI-MS/MS sphingolipid quantification applying the multiple reaction monitoring (MRM) approach. Chromatographic separation was achieved on a 1290 Infinity II HPLC (Agilent Technologies, Waldbronn, Germany) equipped with a Poroshell 120 EC-C8 column (3.0 x 150 mm, 2.7 µm; Agilent Technologies) guarded by a pre-column (3.0 x 5 mm, 2.7 µm) of identical material. The column compartment was maintained at 30°C and the injection volume was 10 µL. A mobile phase system consisting of water (solvent A) and acetonitrile/methanol (1:1, v:v; solvent B), both acidified with 0.1% formic acid, was used for gradient elution at an initial composition of 40:60 (A:B, v:v) and a flow rate of 0.5 mL/min. MS/MS analyses were carried out using a 6495C triple-quadrupole mass spectrometer (Agilent Technologies) operating in the positive electrospray ionization mode (ESI+) [3].The following ion source parameters were set: sheath gas temperature, 375°C; sheath gas flow, 12 L/min of nitrogen; nebulizer pressure, 20 psi; drying gas temperature, 240°C; drying gas flow, 20 L/min of nitrogen; capillary voltage, 4000 V; nozzle voltage, 2000 V; iFunnel high pressure RF voltage, 90 V and iFunnel low pressure RF voltage, 60 V.
The following mass transitions were recorded (qualifier product ions in parentheses): long-chain bases (LCB): *m/z* 300.3 → 282.3 (252.3) for Sph, *m/z* 302.3 → 284.3 (254.3) for dhSph, *m/z* 307.3 → 289.3 (259.3) for d_7-Sph, *m/z* 309.4 → 291.3 (261.3) for d_7-dhSph, *m/z* 380.3 → 264.3 (82.1) for S1P, and *m/z* 387.3 → 271.3 (82.1) for d_7-S1P; ceramides (Cer): *m/z* 520.5 → 264.3 (282.3) for 16:0 Cer, *m/z* 534.5 → 264.3 (282.3) for 17:0 Cer, *m/z* 548.5 → 264.3 (282.3) for 18:0 Cer, *m/z* 576.6 → 264.3 (282.3) for 20:0 Cer, *m/z* 604.6 → 264.3 (282.3) for 22:0 Cer, *m/z* 630.6 → 264.3 (282.3) for 24:1 Cer, and *m/z* 632.6 → 264.3 (282.3) for 24:0 Cer; dihydroceramides (dhCer): *m/z* 540.5 → 522.6 (284.3) for 16:0 dhCer, *m/z* 568.5 → 550.5 (284.3) for 18:0 dhCer, *m/z* 596.6 → 578.6 (284.3) for 20:0 dhCer, *m/z* 624.6 → 606.6 (284.3) for 22:0 dhCer, *m/z* 650.7 → 632.7 (284.3) for 24:1 dhCer, and *m/z* 652.7 → 634.6 (284.3) for 24:0 dhCer; sphingomyelins (SM): *m/z* 703.6 → 184.1 (86.1) for 16:0 SM, *m/z* 731.6 → 184.1 (86.1) for 18:0 SM, *m/z* 734.6→ 184.1 (86.1) for 16:0-d_{31} SM, *m/z* 759.6 → 184.1 (86.1) for 20:0 SM, *m/z* 787.7 → 184.1 (86.1) for 22:0 SM, *m/z* 813.7 → 184.1 (86.1) for 24:1 SM, and *m/z* 815.7 → 184.1 (86.1) for 24:0 SM; hexosylceramides (HexCer): *m/z* 700.6 → 264.2 (682.6, 520.6) for 16:0 HexCer, *m/z* 714.6 → 264.2 (696.6, 534.6) for 17:0 HexCer, and *m/z* 810.7 → 264.2 (792.7, 630.6) for 24:1 HexCer; lactosylceramides (LacCer): *m/z* 862.6 → 264.3 (844.6, 520.5) for 16:0 LacCer, *m/z* 876.6 → 264.3 (858.6, 534.5) for 17:0 LacCer, and *m/z* 972.7 → 264.2 (954.8, 630.7) for 24:1 LacCer.
Peak areas of Cer, dhCer, SM, HexCer and LacCer sub-species, as determined with MassHunter software (Agilent Technologies), were normalized to those of their internal standards (17:0 Cer for Cer and dhCer sub-species; 16:0-d_{31} SM for SM sub-species; 17:0 HexCer for HexCer sub-species and 17:0 LacCer for LacCer sub-species) followed by external calibration in the range of 1 fmol or 10 fmol (HexCer, LacCer) to 5 pmol (dhCer), 15 pmol (HexCer, LacCer) or 50 pmol (Cer, SM) on column. DhSph, Sph and S1P were directly quantified via their deuterated internal standards d_7-dhSph (0.125 pmol on column), d_7-Sph (0.25 pmol on column) and d_7-S1P (0.125 pmol on column). Quantification was performed with MassHunter Quantitative Analysis software (version 10.1, Agilent Technologies).

2. Supplementary references

1. Raasch, M. et al. Microfluidically supported biochip design for culture of endothelial cell layers with improved perfusion conditions. Biofabrication 7, 015013 (2015). https://doi.org/10.1088/1758-5090/7/1/015013

2. Siwczak, F. et al. Human macrophage polarization determines bacterial persistence of Staphylococcus aureus in a liver-on-chip-based infection model. Biomaterials 287, 121632 (2022). https://doi.org/10.1016/j.biomaterials.2022.121632
3. Naser, E. et al. Characterization of the small molecule ARC39, a direct and specific inhibitor of acid sphingomyelinase in vitro. J Lipid Res 61, 896-910 (2020). https://doi.org/10.1194/jlr.RA120000682

2. Supplementary figures and legends

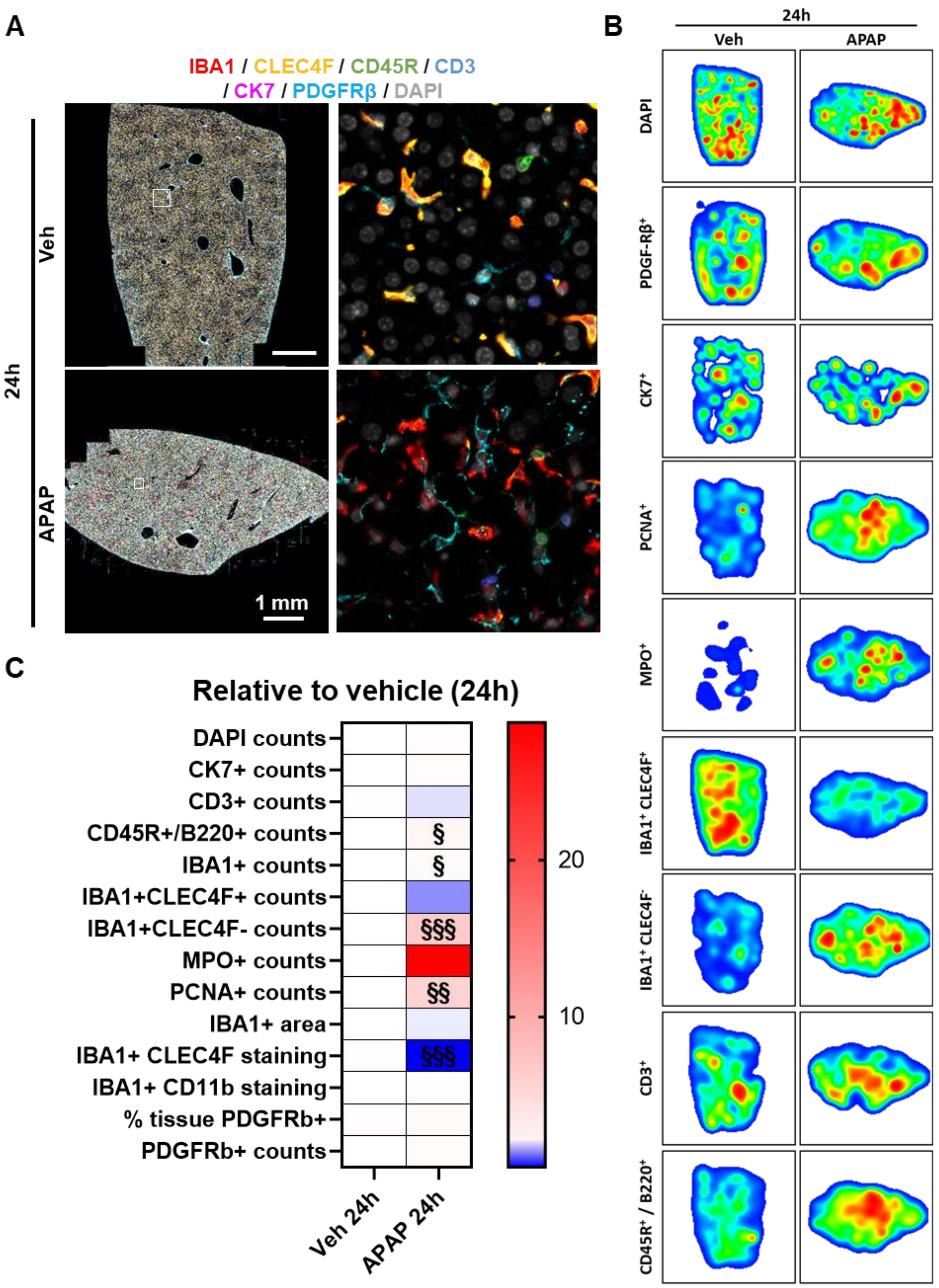
A
IBA1 / CLEC4F / CD45R / CD3
/ CK7 / PDGFRβ / DAPI
24h
Veh
APAP
1 mm
B
24h
Veh
APAP
DAPI
PDGF-Rβ+
CK7+
PCNA+
MPO+
IBA1+ CLEC4F+
IBA1+ CLEC4F-
CD3+
CD45R+ / B220+
C
Relative to vehicle (24h)
DAPI counts
CK7+ counts
CD3+ counts
CD45R+/B220+ counts
IBA1+ counts
IBA1+CLEC4F+ counts
IBA1+CLEC4F- counts
MPO+ counts
PCNA+ counts
IBA1+ area
IBA1+ CLEC4F staining
IBA1+ CD11b staining
% tissue PDGFRb+
PDGFRb+ counts
§
§
§§§
§§
§§§
20
10
Veh 24h
APAP 24h

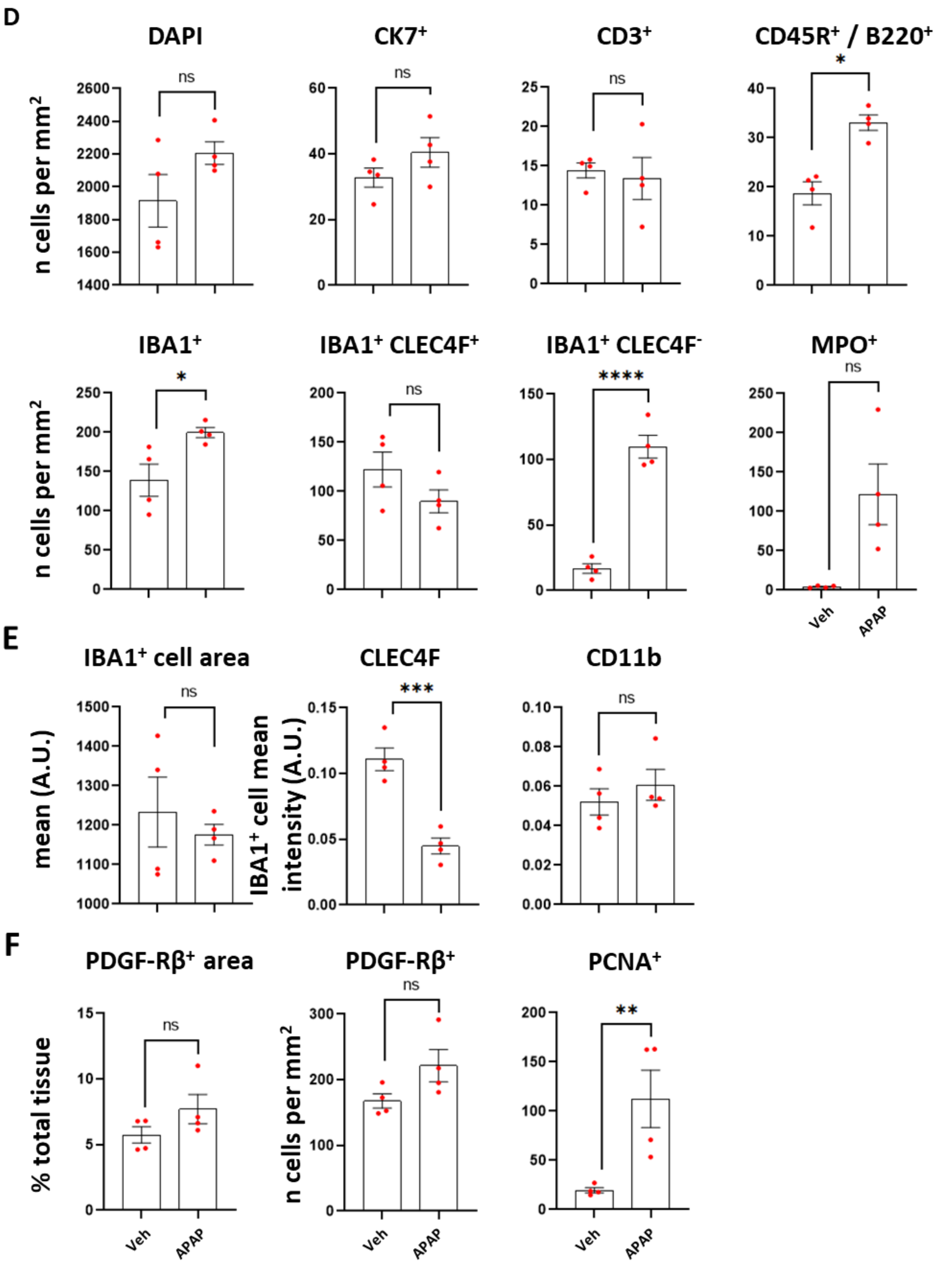

Figure S1. Multiplex immunostaining used to characterize the changing immune landscape in the mouse acute acetaminophen-induced liver injury model. Mice received a single APAP injection and tissues were collected 24 hours later. **(A)** Representative fields of view depicting liver tissue sections subjected to multiplex immunostaining. **(B)** Heat-maps representing cellular density distribution across the samples shown in panel (A). Cell counts and individual cell measurements were collected and are depicted as **(C)** a matrix or **(D-F)** in individual graphs. Sample sizes: n(Veh) = 4, n(APAP) = 4. Abbreviations: APAP:

acetaminophen; Veh: vehicle; CK7: cytokeratin 7; CD3: cluster of differentiation 3; CD45R: cluster of differentiation 45 receptor; IBA1: Ionized calcium binding adaptor molecule 1; CLEC4F: C-Type Lectin Domain Family 4 Member F; MPO: myeloperoxidase; PCNA: proliferating cell nuclear antigen; CD11b: cluster of differentiation 11b; PDGFRB: platelet-derived growth factor receptor beta. One-way ANOVA followed by Student´s t tests was performed. *p<0.05; **p<0.01; ***p<0.005 as compared to vehicle 12h; §p<0.05; §§p<0.01; §§§p<0.005 as compared to vehicle 24h.

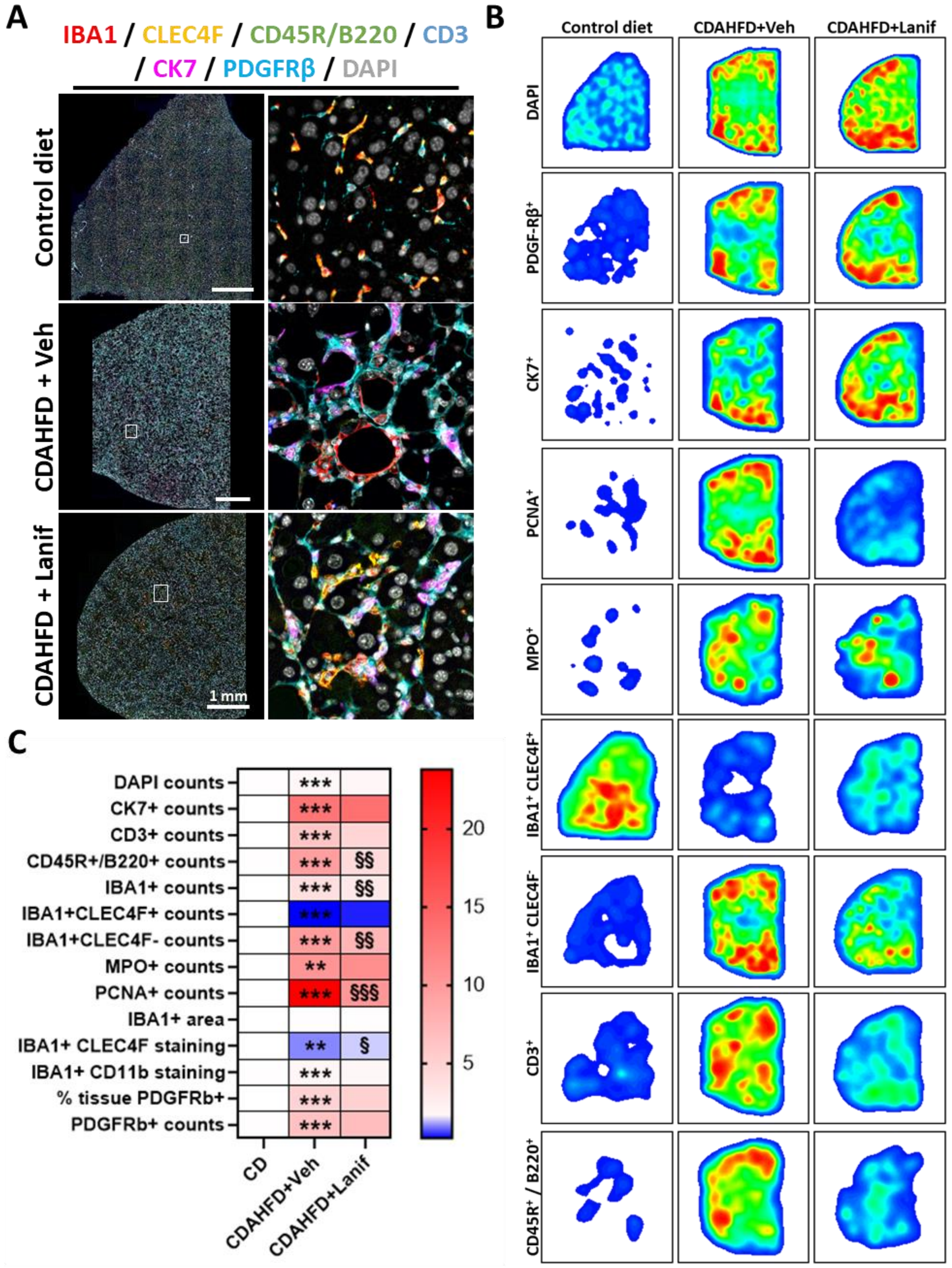

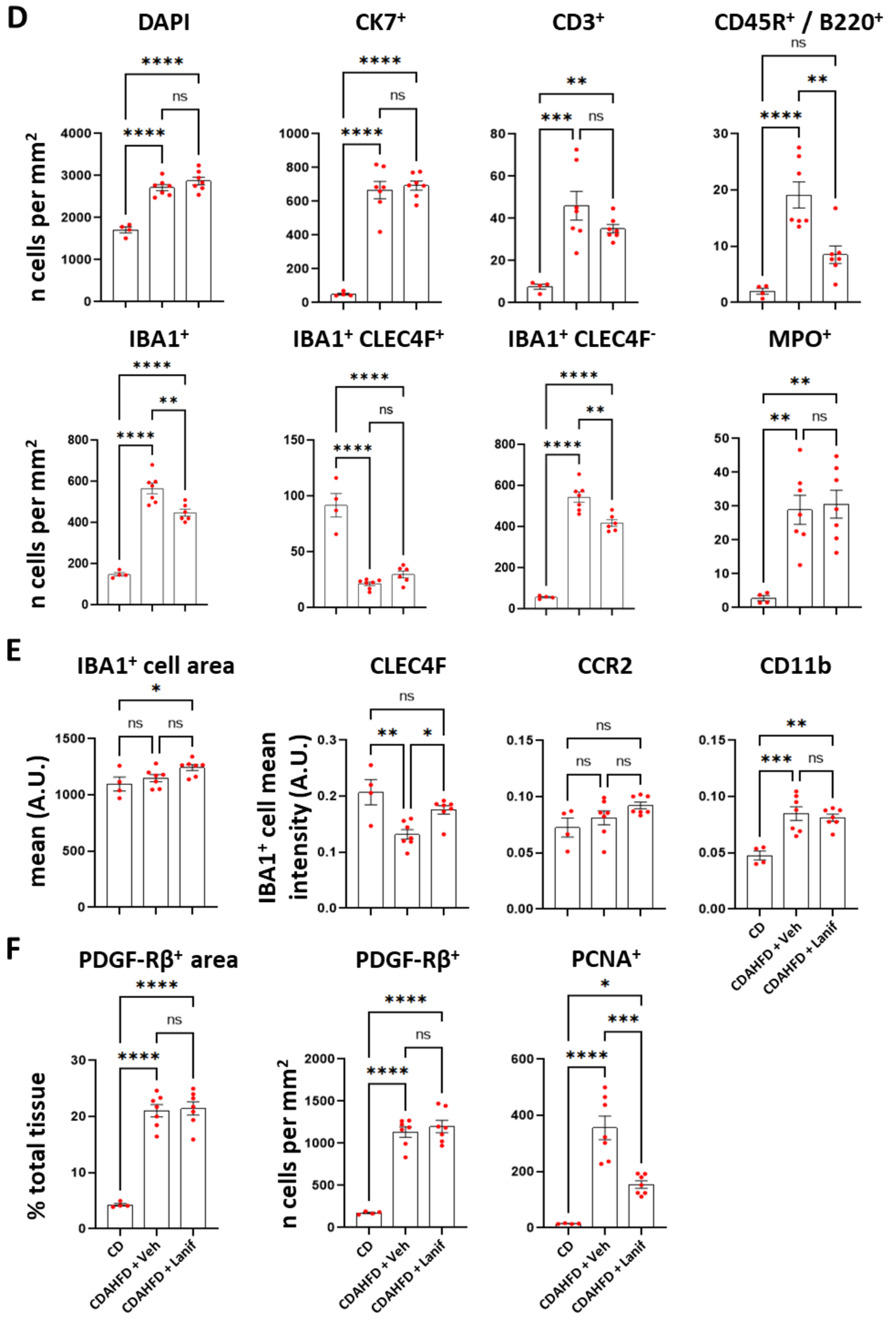

Figure S2. Multiplex immunostaining used to characterize the changing immune landscape in the mouse CDAHFD steatosis model. Mice were fed a CDAHFD diet for 8

weeks and received either vehicle or lanifibranor for the last 2 weeks. **(A)** Representative fields of view depicting liver tissue sections subjected to multiplex immunostaining. **(B)** Heat-maps representing cellular density distribution across the samples shown in panel (A). Cell counts and individual cell measurements were collected and are depicted as **(C)** a matrix or **(D-F)** in individual graphs. **(D)** Individual cell counts normalized to total tissue areas. **(E)** Single cell average size and mean immunostaining intensity for individual markers in IBA1-positive cells. **(F)** PDGF-Rbeta positive areas represented as percentage of total tissue, PDGF-Rbeta- and PCNA-positive cell counts normalized to total tissue areas. Sample sizes: n(control diet) = 4, n(CDAHFD + Veh) = 7, n(CDAHFD + Lanif) = 7. Abbreviations: CDAHFD: choline-deficient L-amino acid-defined, high-fat diet; CD3: cluster of differentiation 3; CD11b: cluster of differentiation 11b; CD45R: cluster of differentiation 45 receptor; IBA1: Ionized calcium binding adaptor molecule 1; CK7: cytokeratin 7; CLEC4F: C-Type Lectin Domain Family 4 Member F; Lanif: lanifibranor; MPO: myeloperoxidase; PCNA: proliferating cell nuclear antigen; PDGF-Rβ: platelet-derived growth factor receptor beta; Veh: vehicle. One-way ANOVA followed by Tukey's multiple comparison was performed. $^{**}p<0.01$; $^{***}p<0.005$ as compared to control diet; §$p<0.05$; §§$p<0.01$; §§§$p<0.005$ as compared to CDAHFD+Veh.

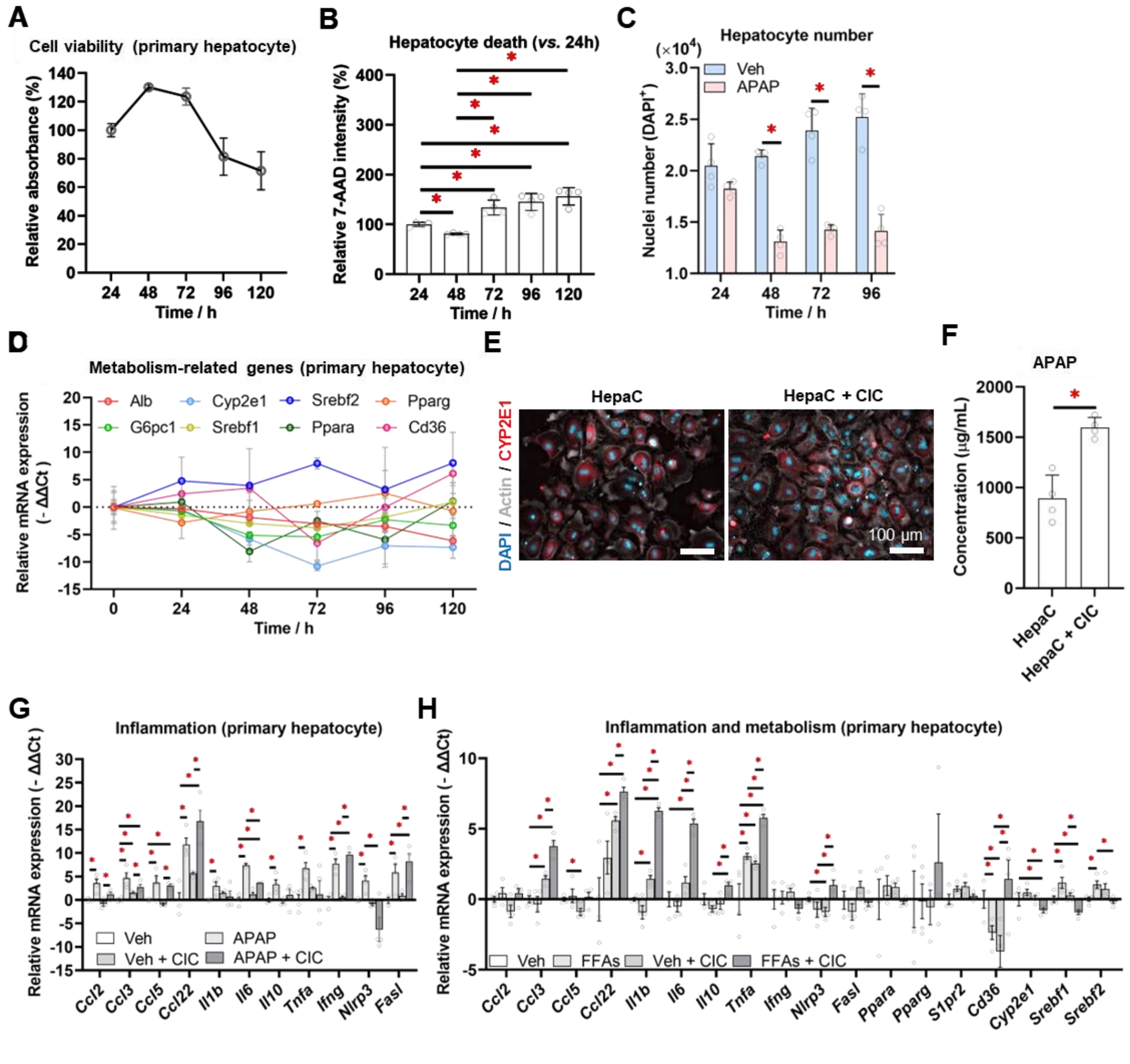

Figure S3. ***In vitro*** **investigations on primary hepatocyte culture with cultivation and gene expression alterations in the presence of CICs. (A)** Relative cell amount of in-vitro

cultivated mouse primary hepatocytes was measured at 0, 24, 48, 72, 96 and 120 h after isolation and seeding. **(B)** Relative cell death levels (7-AAD$^+$) of in-vitro cultivated mouse primary hepatocytes was measured at 0, 24, 48, 72, 96 and 120 h. **(C)** Metabolism-related gene expressions were assessed**. (D)** 7AAD staining was performed upon APAP treatment. (E) CYP2E1 expression was measured on untreated hepatocytes and hepatocytes + CICs. **(F)** Remaining APAP concentration was measured in the culture of hepatocytes and hepatocytes + CICs under APAP treatment for 24 h. **(G, H)** Gene expression analysis of conventional culture systems combining CICs with hepatocytes for 24 h. Sample size: (B-H) n = 4 per group. Abbreviations: *Alb*: albumin; APAP: acetaminophen; *Ccl*: chemokine (C-C motif) ligand; CIC: circulating immune cells; *Cyp2e1*: cytochrome P450 family 2 subfamily E member 1; *Fasl*: FAS ligand; FFAs: free fatty acids; Lanif: lanifibranor; *G6pc1*: glucose-6-phosphatase catalytic subunit 1; *Il*: interleukin; Ifn: interferon; *Nlrp3*: NLR family pyrin domain containing 3; *Ppar*: peroxisome proliferator activated receptor; *S1pr2*: sphingosine-1-phosphate receptor 2; *Srebf*: sterol regulatory element-binding transcription factor; *Tnfa*: tumor necrosis factor-alpha; Veh: vehicle. One-way ANOVA and Tukey's multiple comparison were performed. *p<0.05 as indicated.

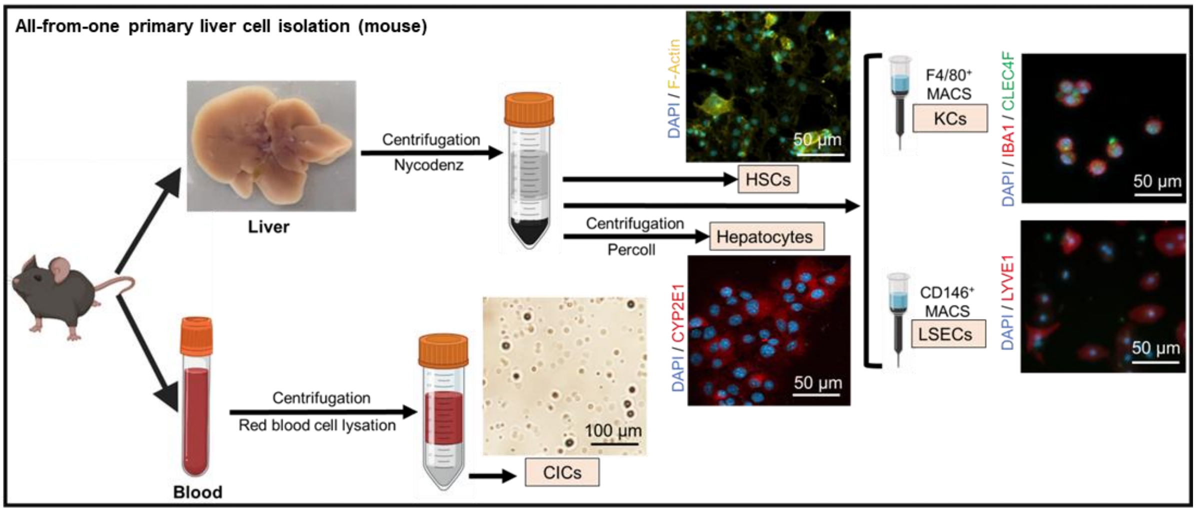

Figure S4. All-from-one primary liver cell isolation (mouse) procedure and multicellular layout of LoC model. (A) Primary hepatocytes, HSCs, KCs and LSECs are sequentially isolated from mouse liver using centrifugation or MACS methods. CICs are isolated from mouse blood using red blood lysis and centrifugation methods. Abbreviations: HSC: hepatic stellate cell; KC: Kupffer cell; LSEC: liver sinusoidal cell; CIC: circulating immune cell; MACS: Magnetic-activated cell sorting.

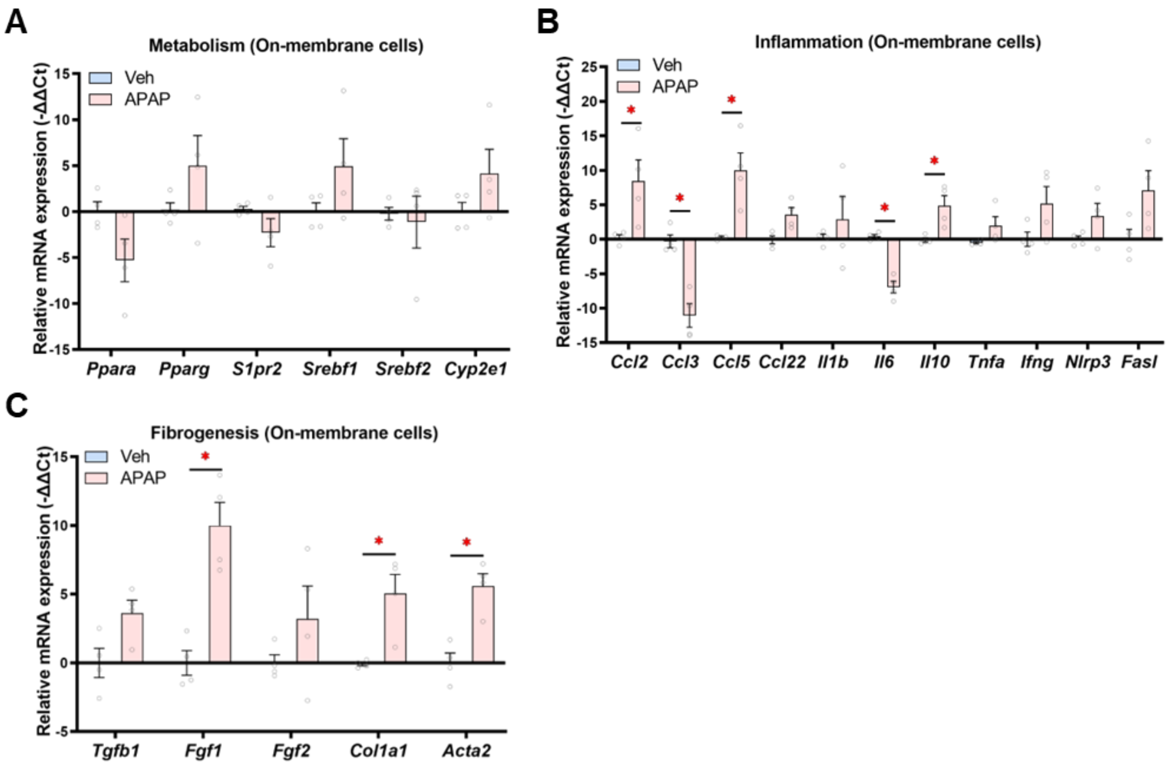

Figure S5. Gene expression profile of on-membrane cells of LoC. (A-C) Gene expression analysis of on-membrane cells of LoC upon APAP administration. Sample size: n = 4 per group. Abbreviations: APAP: acetaminophen; Veh: vehicle; IL: interleukin; CCL: the chemokine (C-C motif) ligand; IFN: interferon; TNF-a: tumor necrosis factor-alpha; NLRP3: NLR family pyrin domain containing 3; FASL: FAS ligand; PPAR: peroxisome proliferator activated receptor; S1PR2: sphingosine-1-phosphate receptor 2; CYP2E1: cytochrome P450 family 2 subfamily E member 1; SREBF: sterol regulatory element-binding transcription factor. Unpaired student's t-tests were performed. *p<0.05 as indicated.

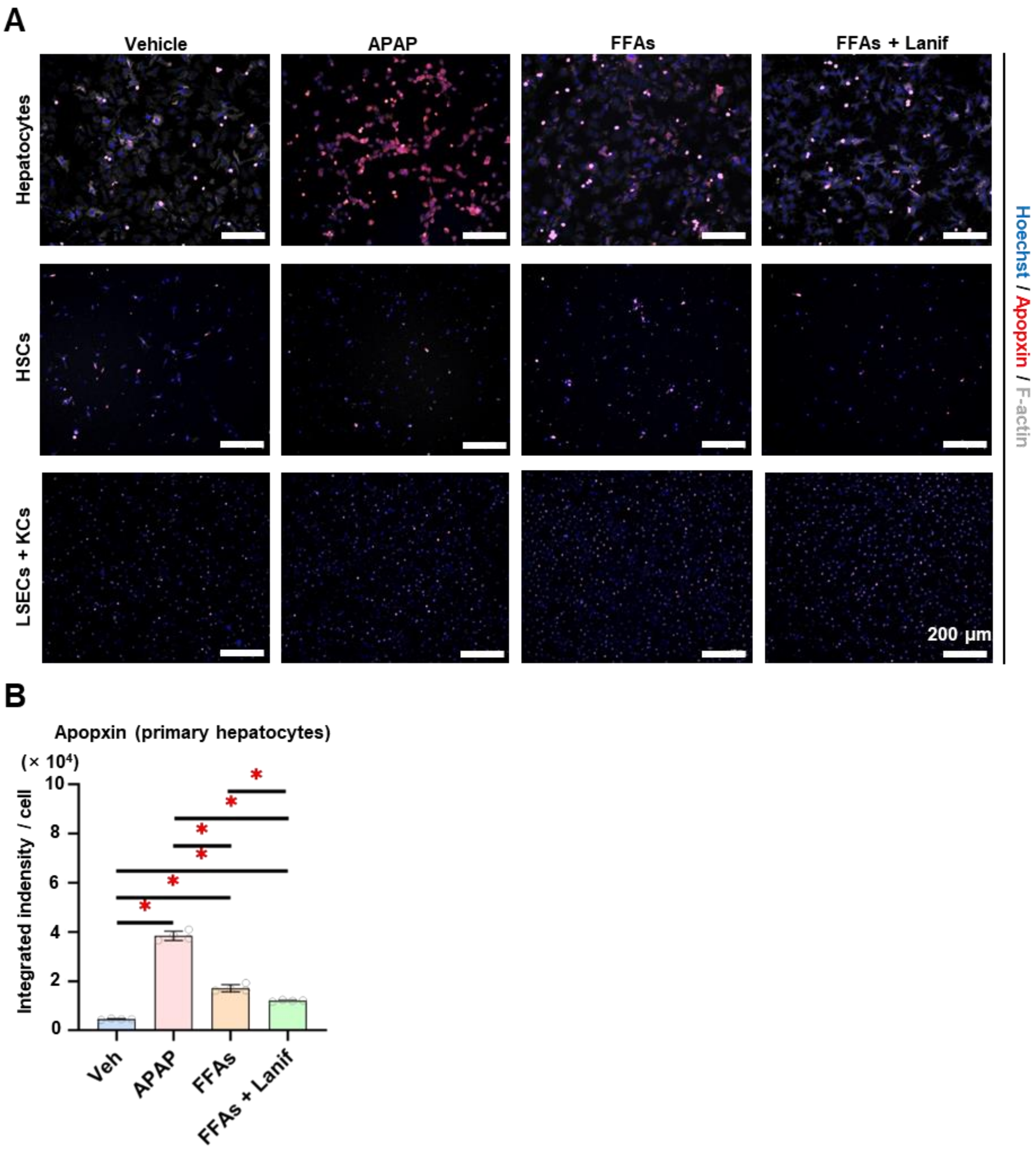
A
Vehicle
APAP
FFAs
FFAs + Lanif
Hepatocytes
HSCs
LSECs + KCs
200 µm
Hoechst / Apopxin / F-actin
B
Apopxin (primary hepatocytes)
(× 10⁴)
Integrated indensity / cell
10
8
6
4
2
0
Veh
APAP
FFAs
FFAs + Lanif

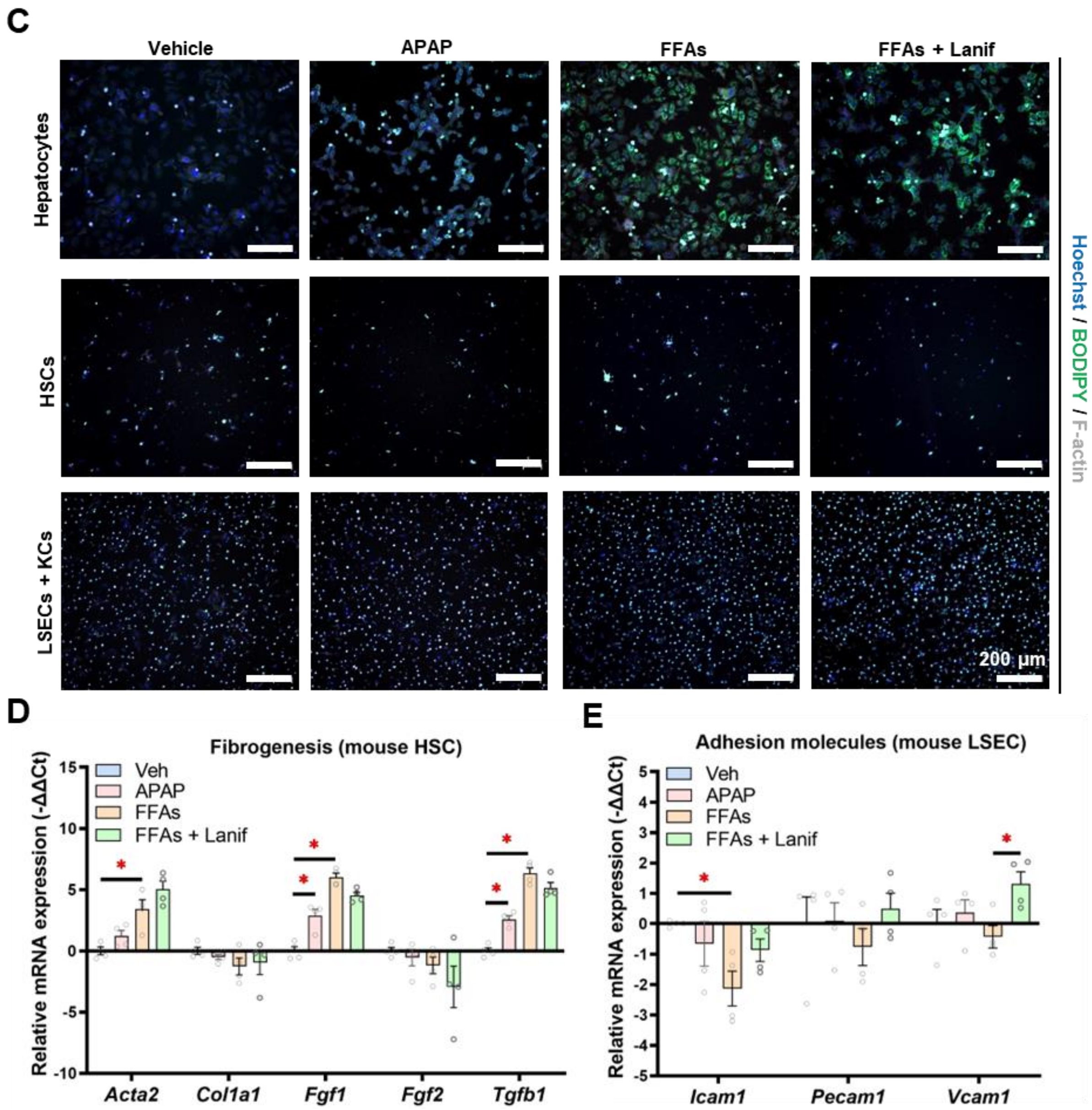

Figure S6. Primary liver cell response to acetaminophen, FFAs and lanifibranor in conventional 2-dimensional culture. Primary liver cells were seeded in conventional 2-dimensional culture and exposed to acetaminophen, FFAs and lanifibranor. **(A)** Cell death was evidenced by Apopxin. **(B)** Apopxin staining in hepatocytes was quantified. **(C)** lipid vesicle accumulation was evidenced by BODIPY. Gene expression was analyzed in **(D)** HSCs and **(E)** LSECs. Sample size: n = 4 per group. Abbreviations: APAP: acetaminophen; FFAs: free fatty acids; HSC: hepatic stellate cell; KC: Kupffer cell; Lanif: lanifibranor; LSEC: liver sinusoidal endothelial cell; Veh: vehicle. Unpaired student’s t-tests or one-way ANOVA and Tukey’s multiple comparison were performed. *$p<0.05$ as indicated.

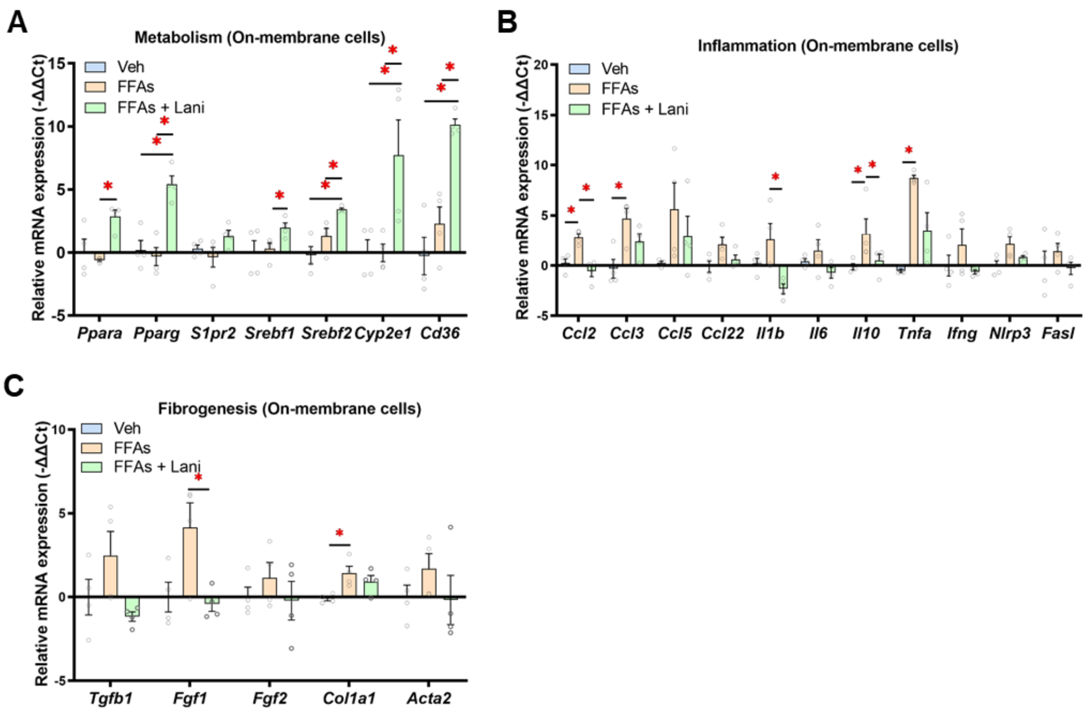

Figure S7. Gene expression profile of on-membrane cells of LoC. (A-C) Gene expression analysis of on-membrane cells of LoC upon FFAs and lanifibranor administration. Sample size: n = 4 per group. Abbreviations: FFAs: free fatty acids; Lanif: lanifibranor; Veh: vehicle; IL: interleukin; CCL: the chemokine (C-C motif) ligand; IFN: interferon; TNF-a: tumor necrosis factor-alpha; NLRP3: NLR family pyrin domain containing 3; FASL: FAS ligand; PPAR: peroxisome proliferator activated receptor; S1PR2: sphingosine-1-phosphate receptor 2; CYP2E1: cytochrome P450 family 2 subfamily E member 1; SREBF: sterol regulatory element-binding transcription factor Unpaired student's t-tests or one-way ANOVA and Tukey's multiple comparison were performed. *p<0.05 as indicated.

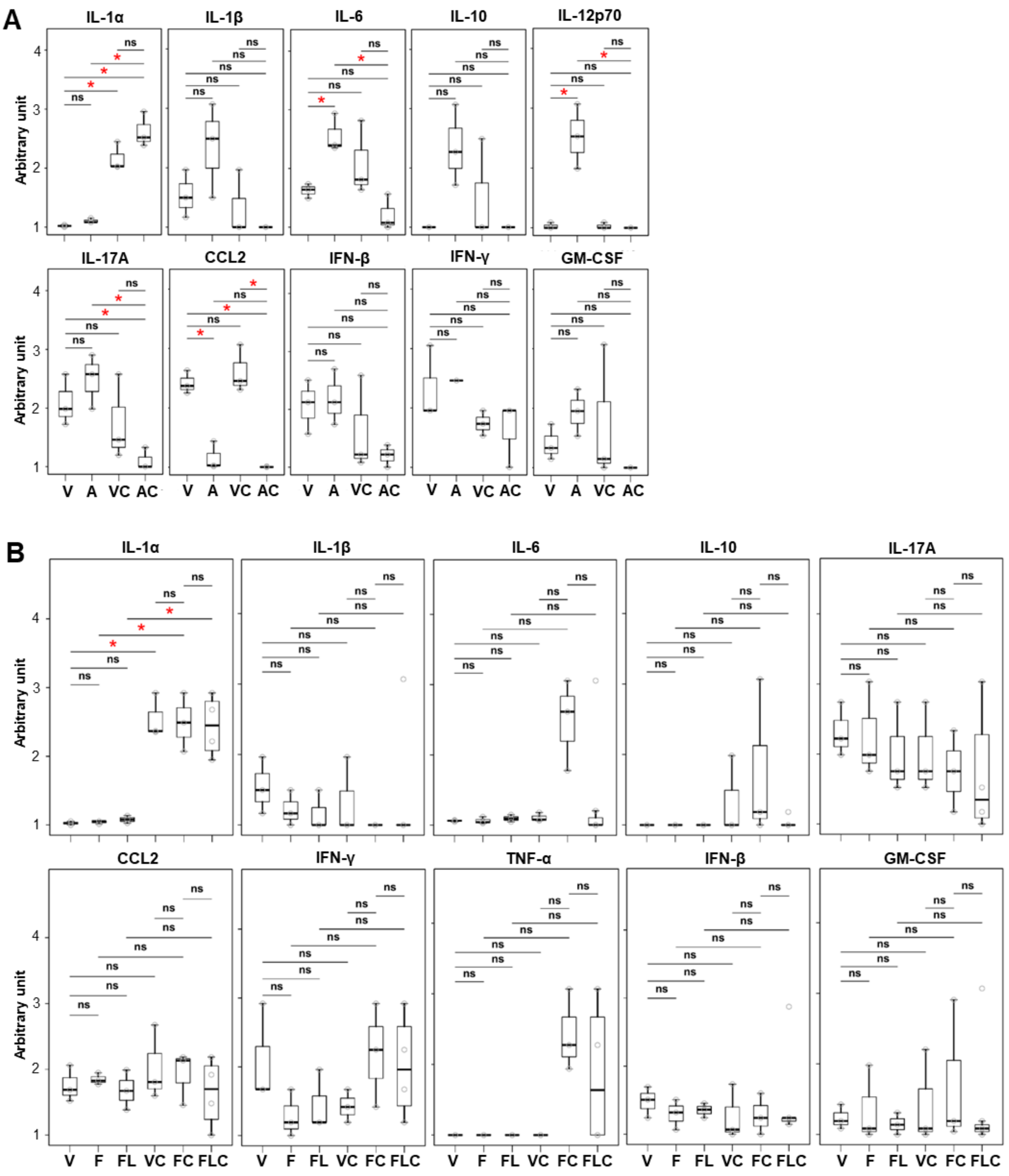

Figure S8. The presence of CICs influences the *in vitro* cytokine milieu upon FFA treatment of primary hepatocytes. Primary mouse hepatocytes were treated with APAP, FFA and lanifibranor in the presence or absence of CICs. Culture medium was collected and cytokine levels were measured. Cytokines levels were measured after **(A)** APAP, or **(B)** FFA and lanifibranor treatments. Data is depicted as arbitrary units due to the low range of the measurements. Sample size: n = 4 per group. Abbreviations: A: acetaminophen; C: circulating immune cells; F or FFAs: free fatty acids; HPLC-ESI-MS/MS: liquid chromatography-electrospray ionization tandem mass spectrometry; L or Lani: lanifibranor; MFI: mean fluorescence intensity; V or Veh: vehicle. One-way ANOVA and Tukey's multiple comparison were performed. *p<0.05 as indicated.

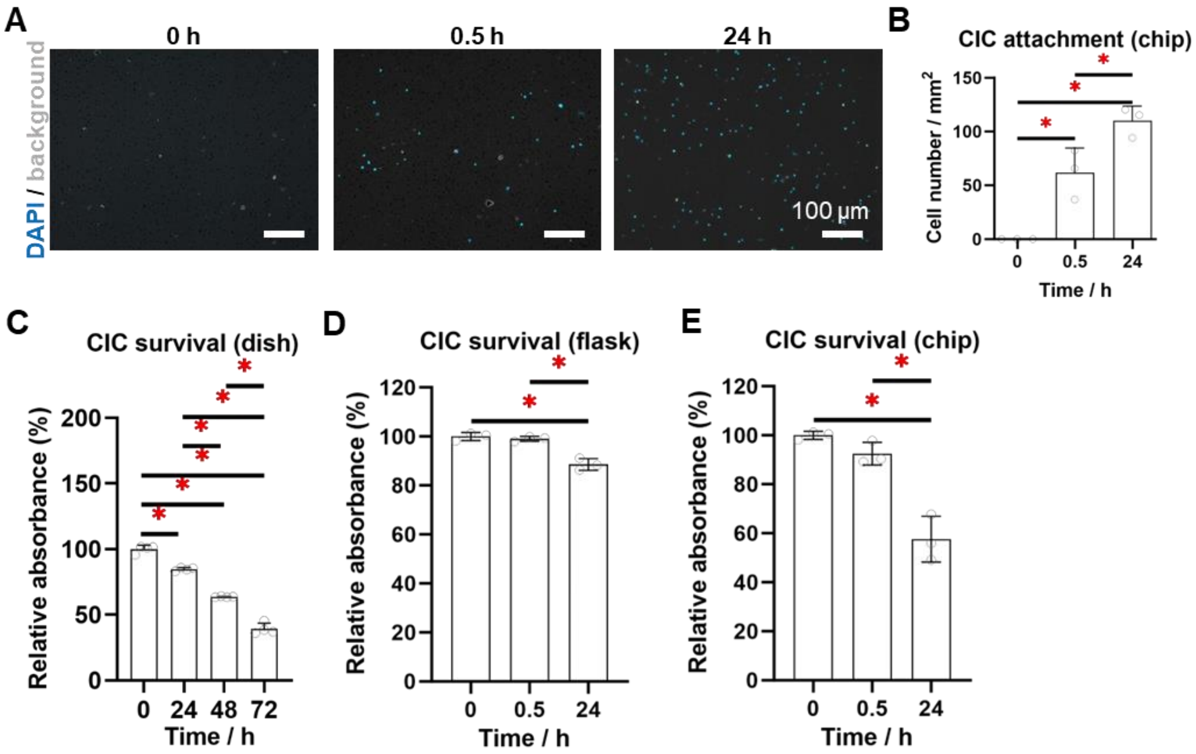

Figure S9. CIC migration to the chip membrane and survival kinetic of primary mouse CICs. CICs were collected from 20-week-old wild-type mice (1 mL blood/mouse) and perfused through a cell-free chip system (CICs of 200 µL blood/chip). CIC accumulating in blank chips were recorded **(A)** and quantified **(B)** at 0, 0.5 and 24 h. **(C)** Survival of CIC cultivated in petri dishes was assessed at 0, 24, 48 and 72 h using the CCK-8 assay. Survival of CIC cultivated in ultra-low-attachment flasks **(D)** or perfused in blank chips **(E)** were assessed at 0, 0.5 and 24 h using the CCK-8 assay. Sample size: (A, B) n = 3 per group, (C-E) n = 4 per group. Abbreviations: CIC: circulating immune cell; LoC: liver-on-a-chip. One-way ANOVA and Tukey's multiple comparison were performed. *p<0.05 as indicated.

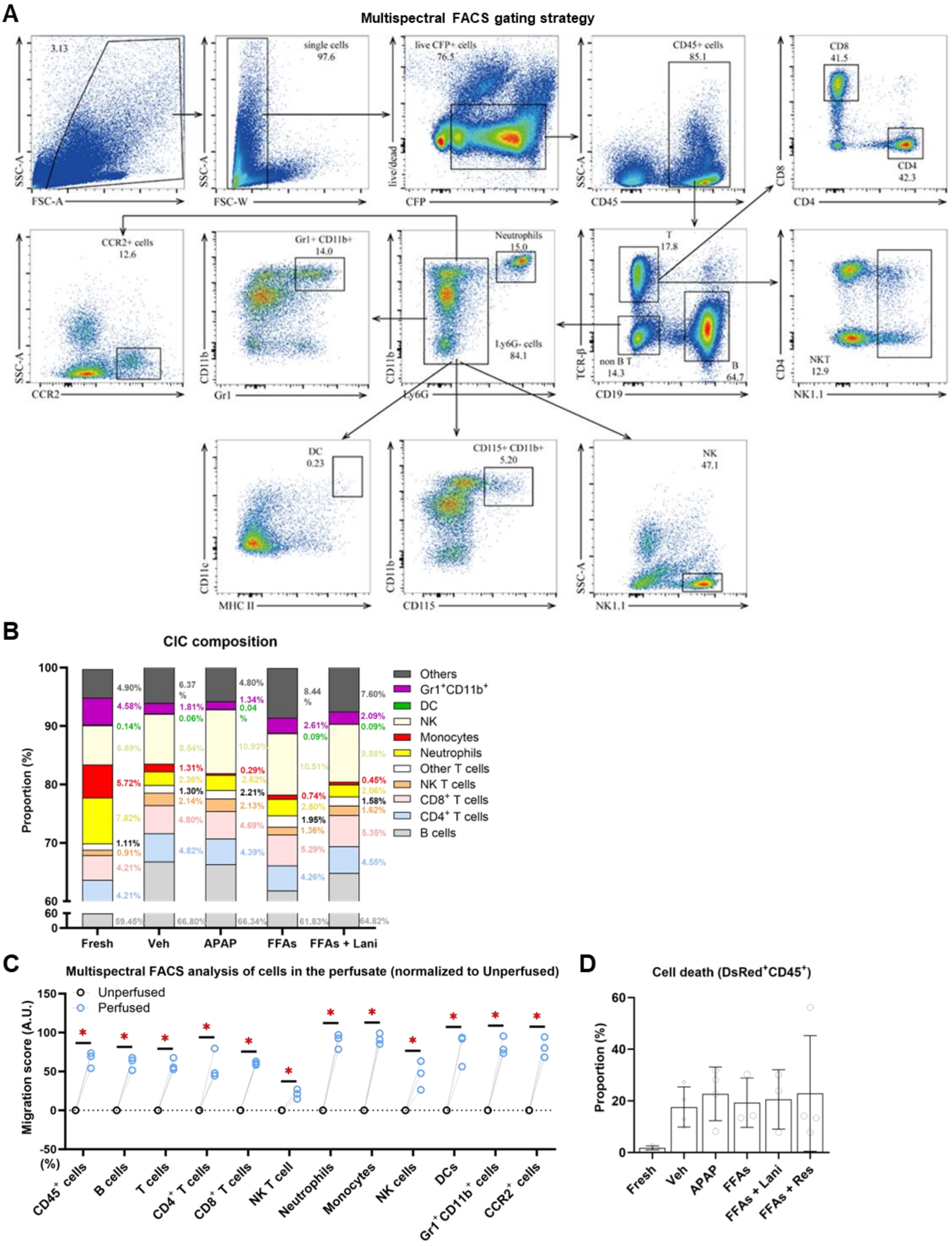

Figure S10. Multispectral flow cytometry to decipher the immune cell migration to the LoC. CICs were isolated from healthy mouse blood and analyzed by flow cytometry either before or after perfusion into the LoC. **(A)** Gating strategy used to identify the distinct immune cell populations. **(B)** Comparison of the relative immune cell composition between fresh (unperfused) or perfused (vehicle, APAP, FFAs and lanifibranor) CICs. **(C)** Migration scores after perfusing fresh CICs into a control LoC. **(D)** Cell death of CICs was assessed on both fresh/unperfused and perfused samples. Sample size: (B, C) n = 3 per group, (D) n = 4

per group. Abbreviations: APAP: acetaminophen; DCs: dendritic cells; FFAs: free fatty acids; Lani: lanifibranor; NK: natural killer. Paired student's t-tests (C) and One-way ANOVA and Tukey's multiple comparison (D) were performed. *p<0.05 as indicated.

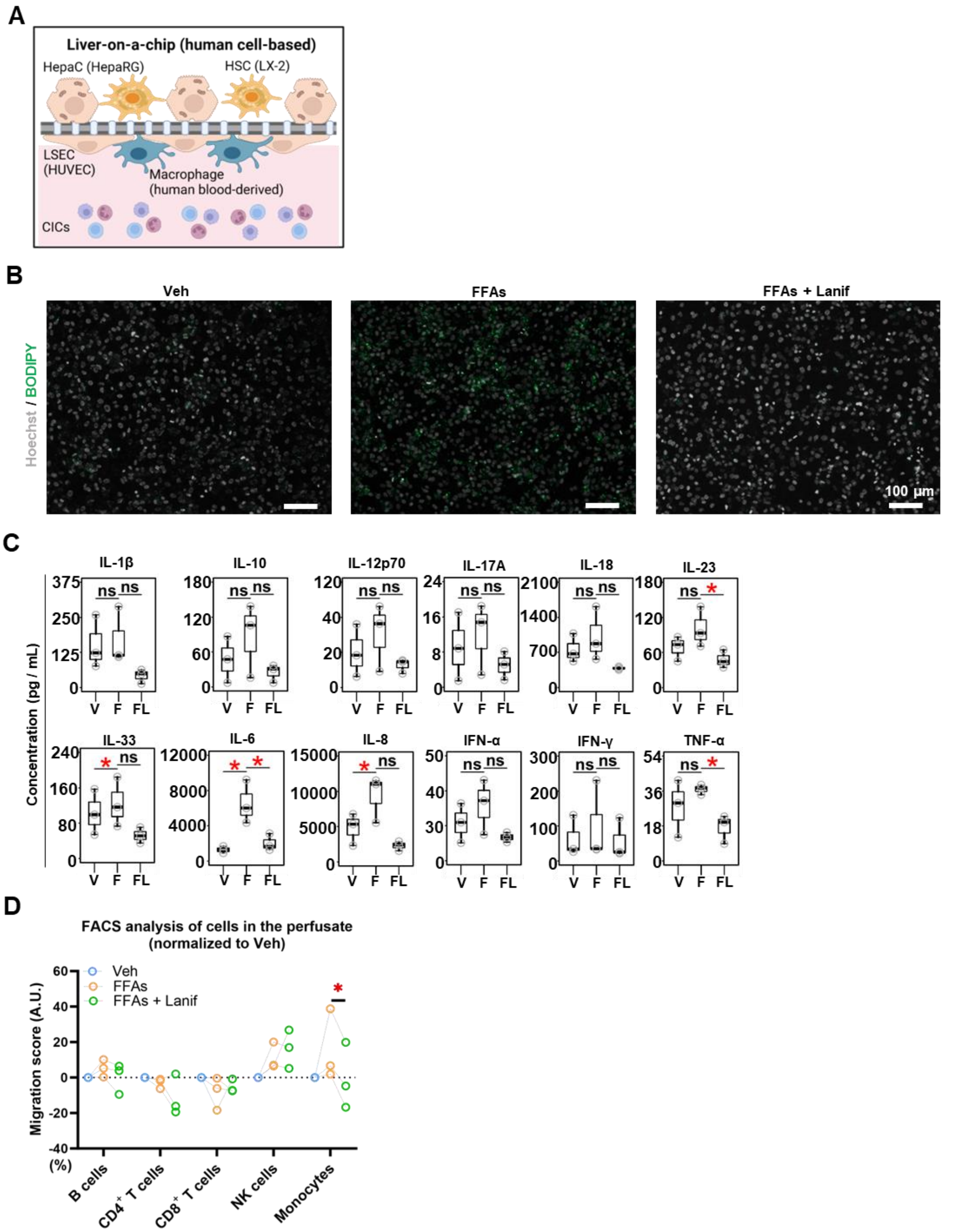

Figure S11. Recapitulation of liver steatosis and therapeutic efficacy in the human LoC. Human cell lines and primary blood cells were isolated and seeded in the human LoC. Liver steatosis and therapeutic efficacy were demonstrated upon vehicle, FFAs and FFAs + lanifibranor treatments. **(A)** Scheme of human LoC. **(B)** Lipid vesicle accumulation in LoC

hepatocytes was verified by BODIPY**.** **(C)** Circulating cytokine levels measured in the perfusate from the LoC exposed to FFAs and lanifibranor. **(D)** CIC depletion from the LoC perfusate after FFA ± lanifibranor treatment is shown for the respective immune cell types. Sample size: n = 3 per group. Abbreviations: F or FFAs: free fatty acids; FL or FFAs + Lanif: free fatty acids plus lanifibranor; FACS: fluorescence-activated cell sorting; IFN: interferon; IL: interleukin; NK: natural killer; TNF-α/γ: tumor necrosis factor-alpha/gamma; V or Veh: vehicle. One-way ANOVA and Tukey's multiple comparison were performed. *p<0.05 as indicated.

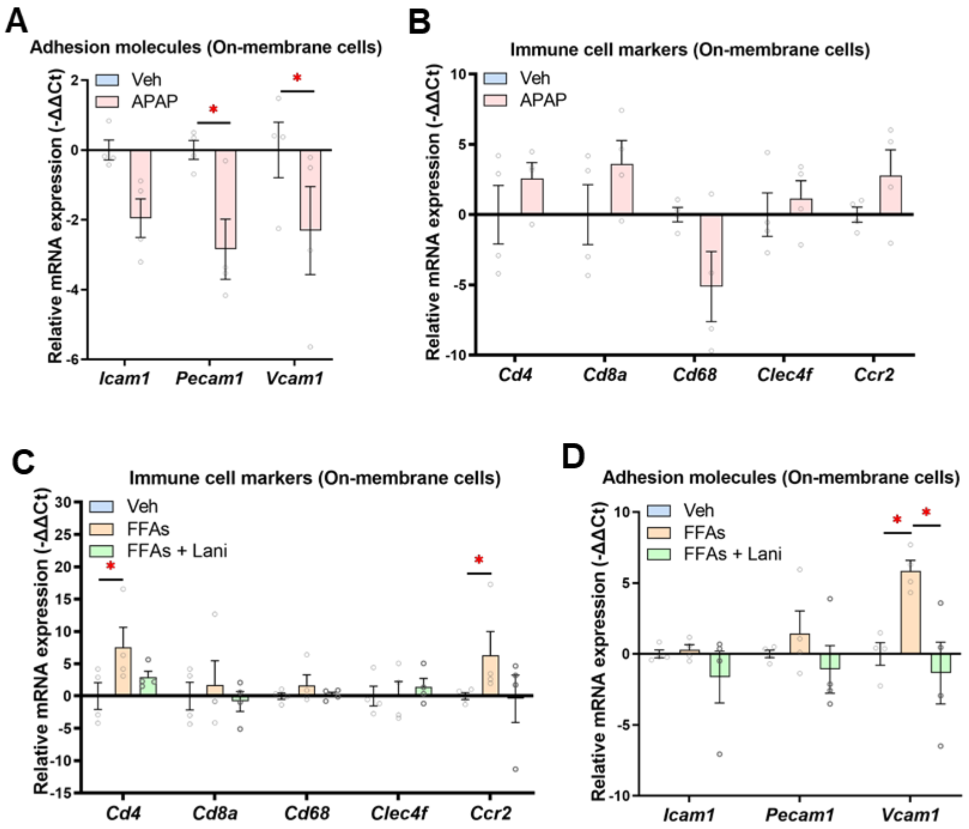

Figure S12. Gene expression analysis to assess the immune cell migration to the LoC. (A-D) Gene expression analysis of on-membrane cells of LoC upon APAP, FFAs and lanifibranor administration. Sample size: n = 4 per group. Abbreviations: APAP: acetaminophen; CIC: circulating immune cell; FFAs: free fatty acids; Lani: lanifibranor; Veh: vehicle. Unpaired student's t-tests were performed.

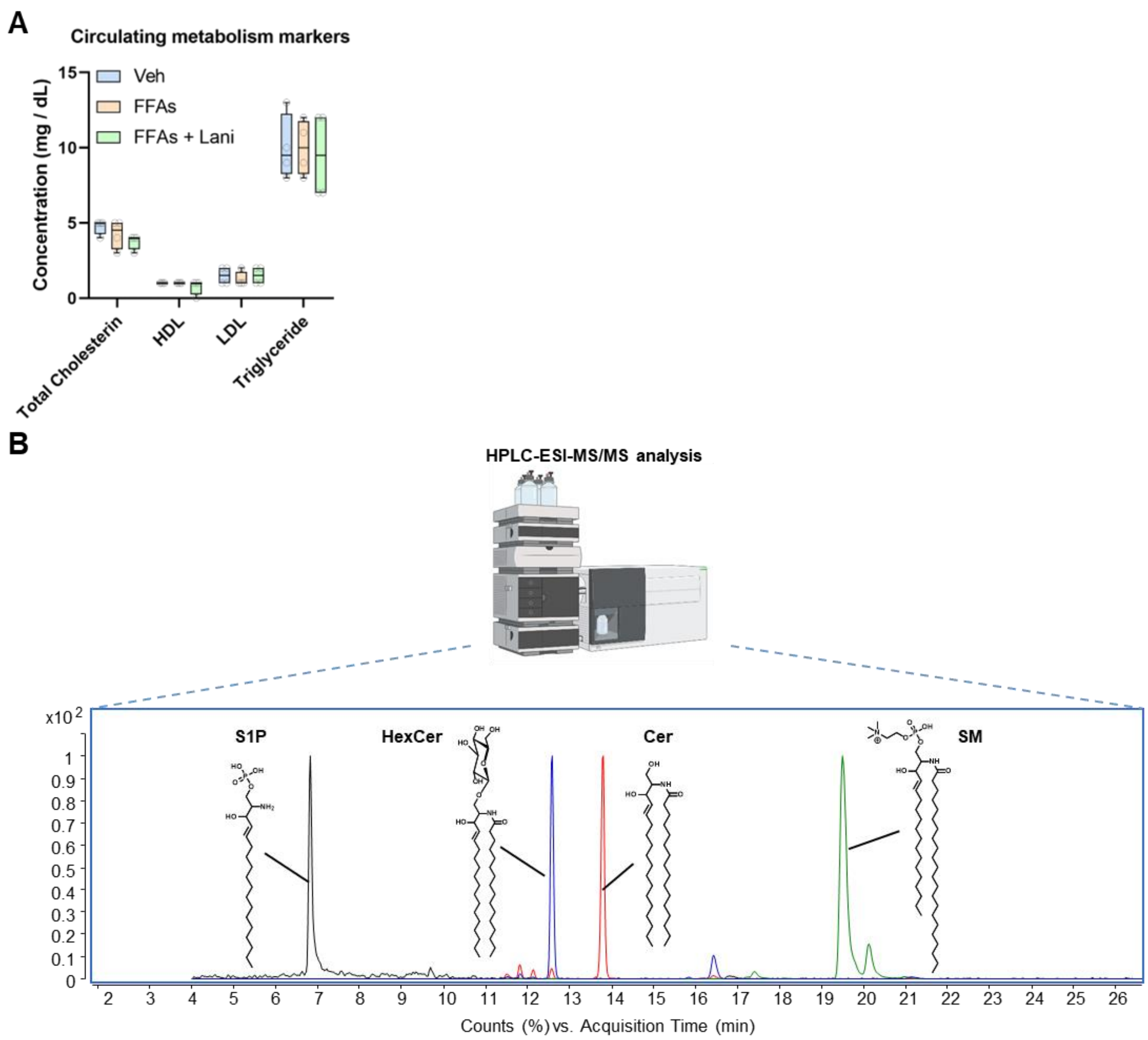

Figure S13. Lipid metabolites measurement in LoC. (A) Concentrations of total cholesterin, HDL-cholesterin, LDL-cholesterin and triglyceride were measured upon APAP, FFAs, and lanifibranor administration. **(B)** Technical procedure of sphingolipid measurement was depicted. Sample size: (A) n = 4 per group. Abbreviations: Res: resmetirom; HDL: high-density lipoprotein; LDL: low-density lipoprotein. One-way ANOVA and Tukey's multiple comparison were performed.

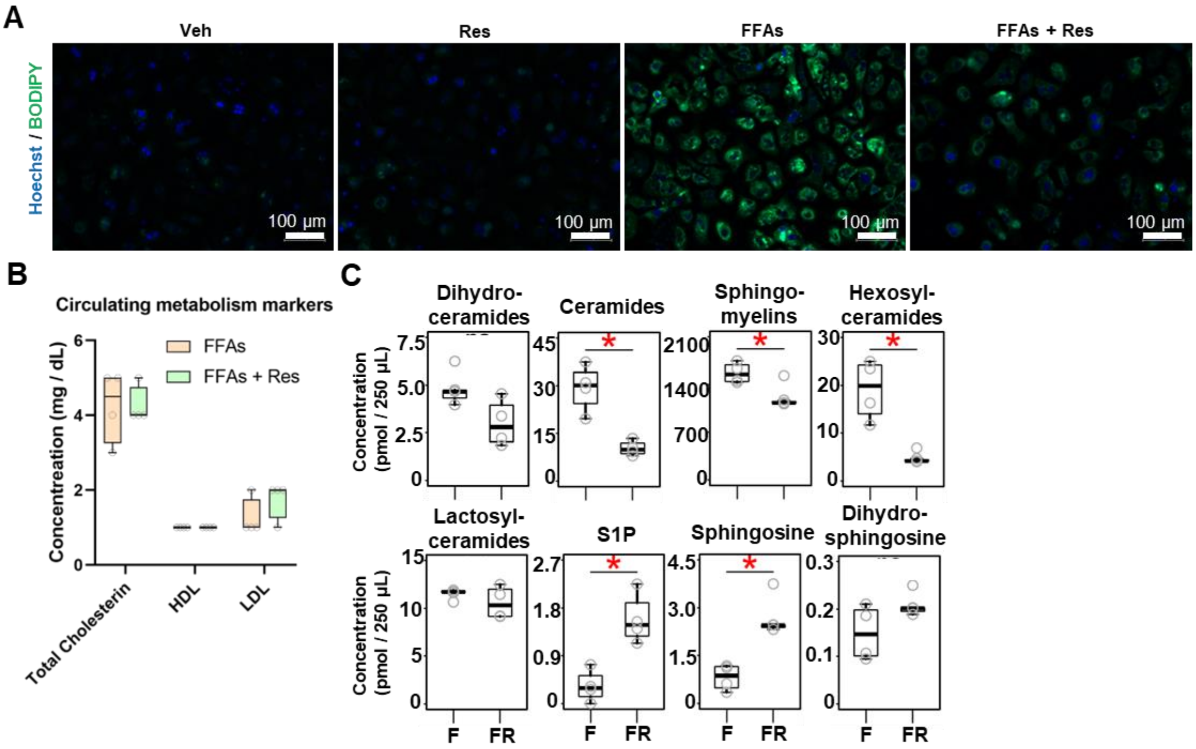
A
Veh
Res
FFAs
FFAs + Res
Hoechst / BODIPY
100 µm
B
Circulating metabolism markers
FFAs
FFAs + Res
Concentreation (mg / dL)
Total Cholesterin
HDL
LDL
C
Dihydro-ceramides
Ceramides
Sphingo-myelins
Hexosyl-ceramides
Lactosyl-ceramides
S1P
Sphingosine
Dihydro-sphingosine
Concentration (pmol / 250 µL)
F
FR

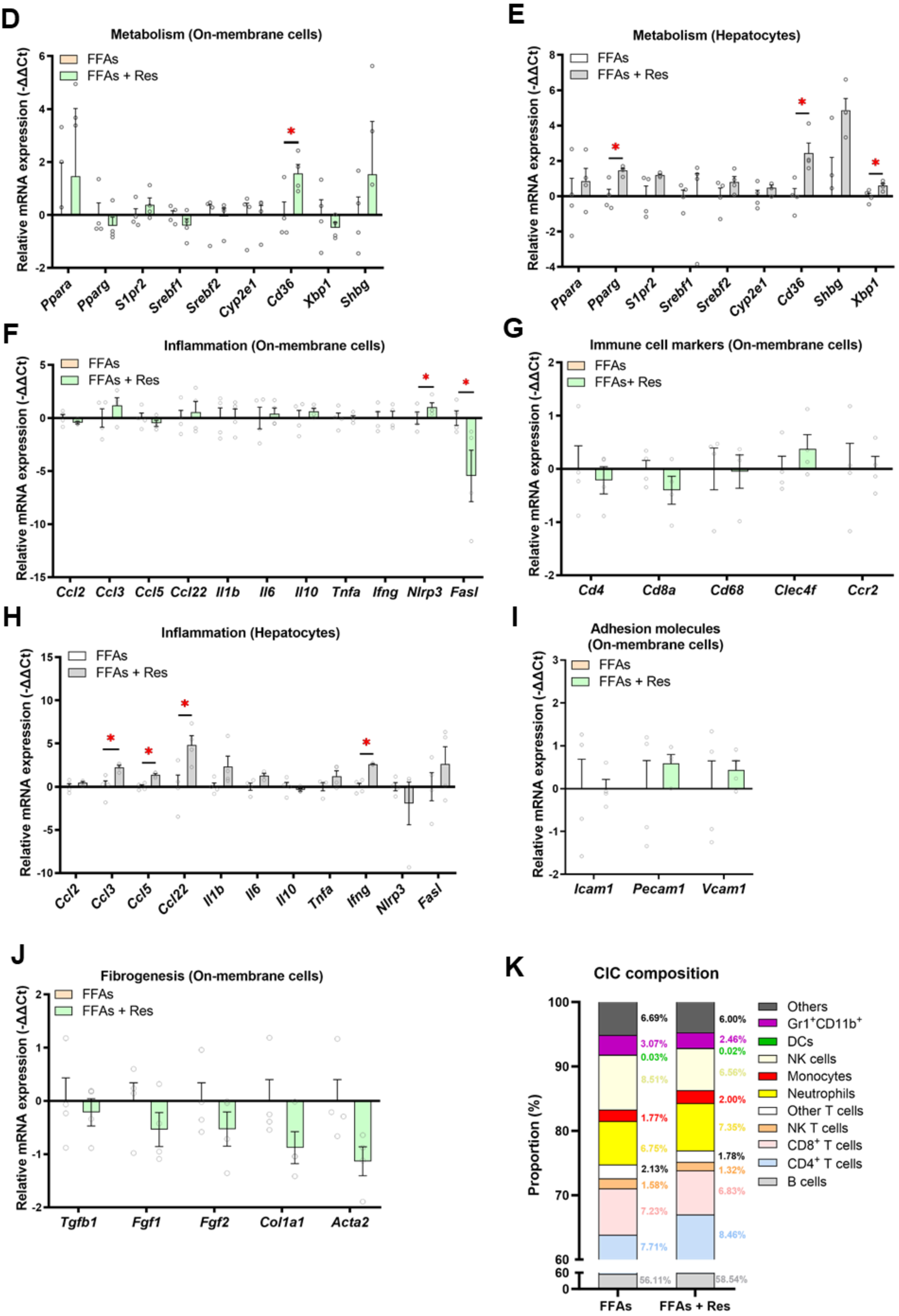
D
Metabolism (On-membrane cells)
FFAs
FFAs + Res
Relative mRNA expression (-ΔΔCt)
Ppara Pparg S1pr2 Srebf1 Srebf2 Cyp2e1 Cd36 Xbp1 Shbg
E
Metabolism (Hepatocytes)
FFAs
FFAs + Res
Relative mRNA expression (-ΔΔCt)
Ppara Pparg S1pr2 Srebf1 Srebf2 Cyp2e1 Cd36 Shbg Xbp1
F
Inflammation (On-membrane cells)
FFAs
FFAs + Res
Relative mRNA expression (-ΔΔCt)
Ccl2 Ccl3 Ccl5 Ccl22 Il1b Il6 Il10 Tnfa Ifng Nlrp3 Fasl
G
Immune cell markers (On-membrane cells)
FFAs
FFAs+ Res
Relative mRNA expression (-ΔΔCt)
Cd4 Cd8a Cd68 Clec4f Ccr2
H
Inflammation (Hepatocytes)
FFAs
FFAs + Res
Relative mRNA expression (-ΔΔCt)
Ccl2 Ccl3 Ccl5 Ccl22 Il1b Il6 Il10 Tnfa Ifng Nlrp3 Fasl
I
Adhesion molecules
(On-membrane cells)
FFAs
FFAs + Res
Relative mRNA expression (-ΔΔCt)
Icam1 Pecam1 Vcam1
J
Fibrogenesis (On-membrane cells)
FFAs
FFAs + Res
Relative mRNA expression (-ΔΔCt)
Tgfb1 Fgf1 Fgf2 Col1a1 Acta2
K
CIC composition
Proportion (%)
6.69% 3.07% 0.03% 8.51% 1.77% 6.75% 2.13% 1.58% 7.23% 7.71% 56.11%
6.00% 2.46% 0.02% 6.56% 2.00% 7.35% 1.78% 1.32% 6.83% 8.46% 58.54%
FFAs FFAs + Res
Others
Gr1+CD11b+
DCs
NK cells
Monocytes
Neutrophils
Other T cells
NK T cells
CD8+ T cells
CD4+ T cells
B cells

Figure S14. Resmetirom effects on primary hepatocyte culture. Primary mouse hepatocytes were cultured in a conventional 2-dimensional system, in the presence or absence of FFAs and resmetirom. Lipid vesicle accumulation was **(A)** visualized by BODIPY. **(B)** Concentrations of total cholesterol, HDL and LDL were measured upon FFAs and resmetirom administration. **(C)** Sphingolipids were measured in the LoC perfusate by HPLC-ESI-MS/MS. **(D-J)** Metabolism, inflammation, adhesion molecule and fibrogenesis-related gene expressions were assessed as indicated, either on the LoC or on conventional hepatocyte cultures. **(K)** Comparison of the relative immune cell composition between FFAs and FFAs + resmetirom. Sample size: n = 4 per group. Abbreviations: FFAs: free fatty acids; Res: resmetirom; Veh: vehicle; ALB: albumin; IL: interleukin; CCL: chemokine (C-C motif) ligand; Ifn: interferon; Tnfa: tumor necrosis factor-alpha; Nlrp3: NLR family pyrin domain containing 3; Fasl: FAS ligand; PPAR: peroxisome proliferator activated receptor; S1PR2: sphingosine 1-phosphate receptor 2; Cyp2e1: cytochrome P450 family 2 subfamily E member 1; Srebf: sterol regulatory element-binding transcription factor. Unpaired student's t-tests were performed. *p<0.05 as indicated.

Supplementary Tables

Table S1. Antibodies, dyes and microbeads

Type	Name	Manufacturer	Catalog No.	Antibody clone	Clone	Host species	Dilution
Primary antibody (ICC/IHC)	Anti-CYP450-2E1	Abcam	ab28146	Polyclonal	-	Rabbit	1/200
	Anti-Cytokeratin-19	DSHB	TROMA-III	Monoclonal	RIgG2a	Rat	1/200
	Anti-CD68	Abcam	ab237968	Monoclonal	FA-11	Rat	1/200
	Anti-LYVE1	Abcam	ab14917	Polyclonal	-	Rabbit	1/200
	Anti-Collagen-I	Abcam	ab270993	Monoclonal	EPR24331-53	Rabbit	1/200
	Anti-CD3	Abcam	ab16669	Monoclonal	SP7	Rabbit	1/500
	Anti-CD11b	Abcam	ab133357	Monoclonal	EPR1344	Rabbit	1/1000
	Anti-CD45R (B220)	BioLegend	103202	Monoclonal	RA3-6B2	Rat	1/200
	Anti-CLEC4F	R&D	MAB2784	Monoclonal	370901	Rat	1/500
	Anti-Cytokeratin-7	Abcam	ab181598	Monoclonal	EPR17078	Rabbit	1/500
	Anti-IBA1	VWR	100369-764	Monoclonal	-	Rabbit	1/500
	Anti-MPO	Abcam	ab208670	Monoclonal	EPR20257	Rabbit	1/1000
	Anti-PCNA	Abcam	ab29	Monoclonal	PC10	Mouse	1/3000
	Anti-PDGF-Rβ	Abcam	ab32570	Monoclonal	Y92	Rabbit	1/200
**Type**	**Name**	**Manufacturer**	**Catalog No.**	**Host species**	**Dilution**		
Secondary antibody (ICC/IHC)	Mouse IgG, Alexa Fluor 555	Cell Signaling	4409S	Goat	1/500		
	Mouse IgG, Alexa Fluor 647	Cell Signaling	4410S	Goat	1/500		
	Rabbit IgG, Alexa Fluor 647	Cell Signaling	4414S	Goat	1/500		
	Rabbit IgG, Alexa Fluor 750	ThermoFisher	A-21039	Goat	1/500		
	Rat IgG, Alexa Fluor 488	Cell Signaling	4416S	Goat	1/500		
	Rat IgG, Alexa Fluor 647	Cell Signaling	4418S	Goat	1/500		
**Type**	**Name**	**Manufacturer**	**Catalog No.**	**Antibody clone**	**Clone**	**Host species**	
Antibodies (Flow cytometry)	CD11b Monoclonal Antibody (M1/70), eFluor™ 450	Invitrogen	48-0112-82	Monoclonal	M1/70	Rat	
	Brilliant Violet 421™ anti-mouse CD4 Antibody	BioLegend	100437	Monoclonal	GK1.5	Rat	
	Brilliant Violet 711™ anti-mouse CD8a Antibody	BioLegend	100747	Monoclonal	53-6.7	Rat	
	FITC anti-mouse Ly-6G Antibody	BioLegend	127605	Monoclonal	1A8	Rat	
	CD45 Monoclonal Antibody (30-F11), Alexa Fluor™ 532	Invitrogen	58-0451-82	Monoclonal	30-F11	Rat	
	PE anti-mouse CD115 (CSF-1R) Antibody	BioLegend	165003	Monoclonal	W19330C	Rat	

	PE/Cyanine5 anti-mouse CD19 Antibody	BioLegend	115509	Monoclonal	6D5	Rat
	PE/Cyanine7 anti-mouse TCR β chain Antibody	BioLegend	109221	Monoclonal	H57-597	Rat
	Alexa Fluor® 700 anti-mouse CD11c Antibody	BioLegend	117319	Monoclonal	N418	Rat
	APC/Cyanine7 anti-mouse NK-1.1 Antibody	BioLegend	108723	Monoclonal	PK136	Rat
	APC/Fire™ 810 anti-mouse Ly-6G/Ly-6C (Gr-1) Antibody	BioLegend	108469	Monoclonal	RB6-8C5	Rat
	PE/Fire™ 810 anti-mouse I-A/I-E Antibody	BioLegend	107667	Monoclonal	M5/114.15.2	Rat
	APC anti-mouse CD192 (CCR2) Antibody	BioLegend	150627	Monoclonal	SA203G11	Rat
	PE Anti-HLA DR + DP + DQ antibody	Abcam	ab23901	Monoclonal	WR18	Mouse
	APC - CD45 Antibody, anti-human, VioGreen™, REAfinity™	Meltenyi	130-110-638	Monoclonal	REA747	Mouse
	BD Horizon™ BV650 Mouse Anti-Human CD3	BD Horizon™	563916	Monoclonal	SP34-2	Mouse
	BD Pharmingen™ FITC Mouse Anti-Human CD4	BD Pharmingen™	561005	Monoclonal	RPA-T4	Mouse
	Brilliant Violet 785™ anti-human CD8 Antibody	BioLegend	344740	Monoclonal	SK1	Mouse
	BD Pharmingen™ APC-Cy™7 Mouse Anti-Human CD19	BD Pharmingen™	561743	Monoclonal	SJ25C1	Mouse
	BD Pharmingen™ PE-Cy™7 Mouse Anti-Human CD56 (NCAM-1)	BD Pharmingen™	557747	Monoclonal	B159	Mouse

Type	Name	Manufacturer	Catalog No.
Cell dyes	Zombie NIR™ Fixable Viability Kit	BioLegend	423105
	Hoechst 33342	Thermo Fisher	62249
	DAPI	Thermo Fisher	D21490
	BODIPY 505/515	Thermo Fisher	D3921
	CellMask plasma membrane stain kit	Thermo Fisher	C37608
	Apoptosis/ Necrosis Assay Kit	Abcam	ab176749

	Oil Red O	Sigma	O0625
**Type**	**Name**	**Manufacturer**	**Catalog No.**
Microbeads (MACS)	Mouse anti-F4/80 Microbeads	Milteny	5210707948
	Mouse anti-CD146 Microbeads	Milteny	5190725032
	Mouse anti-CD326 (EpCAM) Microbeads	Milteny	70285065-00

Type	Target	Manufacturer
Beads (LEGENDplex)	IL-23	BioLegend
	IL-1α	BioLegend
	IFN-γ	BioLegend
	TNF-α	BioLegend
	MCP-1	BioLegend
	IL-12p70	BioLegend
	IL-1β	BioLegend
	IL-10	BioLegend
	IL-6	BioLegend
	IL-8	BioLegend
	IL-27	BioLegend
	IL-33	BioLegend
	IL-17A	BioLegend
	IFN-β	BioLegend
	GM-CSF	BioLegend

Table S2: Primers used in this study.

Supplementary table 2.

Sequences of mouse primers

Genes	Forward 5'-3'	Reverse 5'-3'
Acta2	TGACAGAGGCACCACTGAACC	TCCAGAGTCCAGCACAATACCAGT
Actb	CTCTAGACTTCGAGCAGGAGATGG	ATGCCACAGGATTCCATACCCAAGA
Ccl2	GTG TTG GCT CAG CCA GAT GC	GACACCTGCTGCTGGTGATCC
Ccl22	CCGCAAGCCTGGCGTTGTTTTG	GGGCTTGCTGAGCCTCCCTG
Ccl3	ACCATGACACTCTGCAACCAAG	TCTGCCGGTTTCTCTTAGTCAGG
Ccl5	CTGCTGCTTTGCCTACCTCTCC	GGCACACACTTGGCGGTTCC
Ccr2	TCGCTGTAGGAATGAGAAGAAGAGG	CAAGGATTCCTGGAAGGTGGTCAA
Cd36	TGAATGGTTGAGACCCCGTG	CGTGGCCCGGTTCTACTAAT
Cd4	GGCCAGAGGCTCAGATTC	CAAGCGCCTAAGAGAGATG
Cd68	ACCGCTTATAGCCCAAGGA	GGCAGGGTTATGAGTGACAG
Cd8a	AGTCCTTCAGAAAGTGAACTCTAC	CCGACAATCTTCTGGTCTC
Clec4f	TTTGTGGTGGCTTCACAG	GATTGTCGTGTCCAGAGTTG
Col1a1	TCTGACTGGAAGAGCGGAGAG	GGCACAGACGGCTGAGTAGG
Cyp2e1	ATAGAAGTTGGAACCTGCCC	CTTTGCCAACTTGGTTAAAGAC
Fasl	GTGGCCCATTTAACAGGGAACC	CTCCAGAGATCAGAGCGGTTCC
Fgf1	TTATACGGCTCGCAGACAC	TGCTTCTTGGAGGTGTAAGTG
Fgf2	CGACCCACACGTCAAACTAC	GCACACACTCCCTTGATAGAC
Icam1	CATCACCGTGTATTCGTTTC	GTGAGGTCCTTGCCTACTTG
Ifng	GGAGGAACTGGCAAAAGGATGG	TGTTGCTGATGGCCTGATTGTC
Il10	GGCTGAGGCGCTGTCATCG	TCATTCATGGCCTTGTAGACACC
Il1b	GAGCTGAAAGCTCTCCACCTC	CTTTCCTTTGAGGCCCAAGGC
Il6	GCTACCAAACTGGATATAATCAGGA	CCAGGTAGCTATGGTACTCCAGAA
Nlrp3	TGGGTTCTGGTCAGACACGAG	GGGGCTTAGGTCCACACAGAAA
Pecam1	GAAGGTGCATGGCGTATC	TTCTTGCAAGGAACAATTGAC
Ppara	TGCCTTCCCTGTGAACTGAC	TTCGCCGAAAGAAGCCCTTA
Pparg	ACCTCTGCTGGGGATCTGAA	GCCCAAACCTGATGGCATTG
S1pr2	CAACTCCGGGACATAGACCG	CATGGGGCTGAGCACTGG
Shbg	CCTCACCAAAATCAGCAAACCC	CCCCCTAGTGCAATCCTCAT
Srebp1c	GGAGCCATGGATTGCACATT	GCTTCCAGAGAGGAGGCCAG
Srebp2	CCCTTGACTTCCTTGCTGCA	GCGTGAGTGTGGGCGAATC
Tgfb1	GCTCGCTTTGTACAACAGCACC	GCGGTCCACCATTAGCACG
Tnfa	ACCACGCTCTTCTGTCTACTGA	TCCACTTGGTGGTTTGCTACG
Vcam1	TGTTTGCAGTCTCTCAAGC	GGCTGTCTATCTGGGTTCTC
Xbp1	GAAAGCGCTGCGGAGGAAA	CATAGTCTGAGTGCTGCGGA
